# Genomewide analysis of the lateral organ boundaries domain gene family in *Eucalyptus grandis* reveals members that differentially impact secondary growth

**DOI:** 10.1111/pbi.12754

**Published:** 2017-07-25

**Authors:** Qiang Lu, Fenjuan Shao, Colleen Macmillan, Iain W. Wilson, Karen van der Merwe, Steven G. Hussey, Alexander A. Myburg, Xiaomei Dong, Deyou Qiu

**Affiliations:** ^1^ State Key Laboratory of Tree Genetics and Breeding The Research Institute of Forestry Chinese Academy of Forestry Beijing China; ^2^ CSIRO Agriculture and Food Canberra ACT Australia; ^3^ Department of Genetics, Forestry and Agricultural Biotechnology Institute (FABI) Genomics Research Institute (GRI) University of Pretoria Pretoria South Africa; ^4^ State Key Laboratory of Agrobiotechnology and National Maize Improvement Center Department of Plant Genetics and Breeding China Agricultural University Beijing China

**Keywords:** *Eucalyptus grandis*, lateral organ boundaries domain, gene expression, secondary growth, xylem development, fibre production

## Abstract

Lateral Organ Boundaries Domain (LBD) proteins are plant‐specific transcription factors playing crucial roles in growth and development. However, the function of LBD proteins in *Eucalyptus grandis* remains largely unexplored. In this study, *LBD* genes in *E. grandis* were identified and characterized using bioinformatics approaches. Gene expression patterns in various tissues and the transcriptional responses of *EgLBDs* to exogenous hormones were determined by qRT‐PCR. Functions of the selected *EgLBDs* were studied by ectopically overexpressing in a hybrid poplar (*Populus alba* × *Populus glandulosa*). Expression levels of genes in the transgenic plants were investigated by RNA‐seq. Our results showed that there were forty‐six *EgLBD* members in the *E. grandis* genome and three *EgLBDs* displayed xylem‐ (*EgLBD29*) or phloem‐preferential expression (*EgLBD22* and *EgLBD37*). Confocal microscopy indicated that EgLBD22, EgLBD *2*9 and EgLBD37 were localized to the nucleus. Furthermore, we found that *EgLBD22*,* EgLBD29* and *EgLBD37* were responsive to the treatments of indol‐3‐acetic acid and gibberellic acid. More importantly, we demonstrated *EgLBDs* exerted different influences on secondary growth. Namely, *35S::EgLBD37* led to significantly increased secondary xylem, *35S::EgLBD29* led to greatly increased phloem fibre production, and *35S::EgLBD22* showed no obvious effects. We revealed that key genes related to gibberellin, ethylene and auxin signalling pathway as well as cell expansion were significantly up‐ or down‐regulated in transgenic plants. Our new findings suggest that *LBD* genes in *E. grandis* play important roles in secondary growth. This provides new mechanisms to increase wood or fibre production.

## Introduction

The Lateral Organ Boundaries Domain (LBD) proteins, also known as ASYMMETRIC LEAVES2‐LIKE (ASL) proteins, are a family of plant‐specific transcription factors with a highly conserved Lateral Organ Boundaries (LOB) Domain (Iwakawa *et al*., 2002). The first *LBD* gene was identified in *Arabidopsis thaliana* based on the distinctive gene expression pattern of an enhancer trap insertion (Shuai *et al*., 2002). The *LBD* gene family of *A. thaliana* can be divided into two classes according to the structure of the LOB domain. Class I have a completely conserved CX_2_CX_6_CX_3_C zinc finger‐like motif, GAS (Gly‐Ala‐Ser) block and an LX_6_LX_3_LX_6_L leucine zipper‐like coiled‐coil motif, whereas class II only contain a conserved zinc finger‐like motif (Iwakawa *et al*., 2002; Shuai *et al*., 2002). The zinc finger‐like motif is thought to be required for DNA binding, and the leucine zipper‐like motif presumably participates in protein dimerization (Majer and Hochholdinger, 2011; Matsumura *et al*., 2009).

The *LBD* gene families of Arabidopsis, rice, poplar, tomato, apple, Medicago*,* maize, grape, mulberry and barley has been studied with members ranging from 24 to 58 (Cao *et al*., 2016; Jia *et al*., 2014; Luo *et al*., 2016; Shuai *et al*., 2002; Wang, 2016; Wang *et al*., 2013a,b; Yang *et al*., 2006; Zhang *et al*., 2014; Zhu *et al*., 2007). The *LBD* genes are found only in plants, implying that this gene family may regulate plant‐specific growth and development processes (Shuai *et al*., 2002). Previous studies of the *LBD* gene family in various plant species have shown that they play an important role in many developmental processes, including leaf, lateral root, inflorescence, embryo and flower development (Borghi *et al*., 2007; Bortiri *et al*., 2006; Liu *et al*., 2005; Xu *et al*., 2003, 2008). In addition, *LBD* genes were also involved in plant secondary metabolism, assimilation of nitrogen nutrition as well as hormone‐mediated plant lateral organ development (Albinsky *et al*., 2010; Bell *et al*., 2012; Rubin *et al*., 2009).

Secondary growth results from cell division in the vascular cambium or lateral meristem, causing the stems and roots to thicken, and in woody plants, this process produces secondary xylem inward and secondary phloem outward (Mellerowicz *et al*., 2001; Spicer and Groover, 2010). This event is the engine of wood production and thus of substantial economic interest. Several reports have demonstrated that *LBD* genes play a significant role in control of secondary growth. Soyano *et al*. (2008) revealed that overexpression of *AtLBD30* (*AtASL19*) and *AtLBD18* (*AtASL20*) induced transdifferentiation of cells from nonvascular tissues into tracheary element‐like cells, the basic units that constitute xylem vessels. By analysing the expression patterns of *Populus tremula* × *Populus alba* (*Pta*) *LBD* gene family, Yordanov *et al*. (2010) showed that *PtaLBD1* and *PtaLBD4* were specifically expressed in secondary phloem, while *PtaLBD15* and *PtaLBD18* were preferentially expressed in secondary xylem. Additionally, they found that overexpression of *PtaLBD1* resulted in significantly enhanced secondary phloem production due to up‐regulation of the *Populus* putative ortholog of *ALTERED PHLOEM DEVELOPMENT* (*APL*), a MYB transcription factor‐encoding gene that played a role in specifying phloem identity in Arabidopsis (Bonke *et al*., 2003). However, no attention has been paid to the question whether different *LBD* genes play different roles in secondary growth and what are the mechanisms.

*Eucalyptus grandis* is an important woody plant within the *Eucalyptus* genus that encompasses some of the fastest growing plantation forest species. Its wood is widely used in lignocellulosic biofuel production, paper, pulp and raw cellulose products (Carroll and Somerville, 2009; Rockwood *et al*., 2008). A previous study has proved that members of *PtaLBD* genes play an essential role in control of secondary woody growth (Yordanov *et al*., 2010). However, the function of the *LBD* genes in *E. grandis* remains largely unexplored. Therefore, identification and characterization of *LBD* genes in *E. grandis* is vital in understanding their roles in regulating secondary growth and thus is crucial to improving its wood quality.

In this article, we used various in silico approaches to identify and characterize *E. grandis LBDs*. We identified 46 *EgLBD* genes in *E. grandis* and performed phylogenetic analysis of all EgLBD proteins in this species. We then analysed of their gene structures, conserved domains and subcellular localizations. Moreover, we used qRT‐PCR to study the gene expression patterns in various tissues and the transcriptional responses of *EgLBD* genes to hormone treatments. Finally, we characterized the functions of selected *LBD* genes by ectopic overexpression in a hybrid poplar (*Populus alba* × *Populus glandulosa*). We demonstrate that three *LBD* members (*EgLBD22, EgLBD29* and *EgLBD37*) had different effects on secondary growth and several key genes related to gibberellin, ethylene, auxin signalling pathway, as well as cell expansion, were significantly up‐regulated or down‐regulated in *EgLBD22‐oe*,* EgLBD29‐oe* and *EgLBD37‐oe* plants. Our results demonstrate that manipulating particular *EgLBD* genes can result in large increases in secondary growth, wood formation or fibre production. These findings provide new insights into the mechanisms by which *LBD* genes control secondary growth in trees.

## Results

### Identification, sequence features and phylogeny of the *EgLBD* gene family

To identify *LBD* genes in *E. grandis*, we performed a genomewide prediction of *EgLBD* genes by BLAST analysis of 43 *AtLBDs*, 57 *PtLBDs* and 35 *OsLBDs* against the *E. grandis* genome using the tBLASTn algorithm, SMART and Pfam tools. A total of 46 *LBD* genes from *E. grandis* were identified (Table S2) and numbered according to genome location. The open reading frames (ORFs) of *EgLBD* genes ranged from 213 bp (*EgLBD15*) to 936 bp (*EgLBD21* and *EgLBD38*), the predicted molecular weight varied from 7.6 kDa (*EgLBD15*) to 33.3 kDa (*EgLBD30*) and the theoretical pI varied from 5.03 (*EgLBD26*) to 10.72 (*EgLBD40*) (Table S3).

Analysis of the protein domains within the 46 EgLBDs revealed that 38 EgLBDs belonged to class I, and 8 EgLBDs to class II (Figure S1). Class I contain a completely conserved CX_2_CX_6_CX_3_C zinc finger‐like motif, GAS (Gly‐Ala‐Ser) block and an LX_6_LX_3_LX_6_L leucine zipper‐like coiled‐coil motif, but class II only have a conserved zinc finger‐like motif. The GAS block is 46 amino acids in length, beginning with a FX_2_VH motif and ending with a DP(V/I)YG motif. The Pro residue in the DP(V/I)YG signature is present in all class I proteins in Arabidopsis, but this is not the case in *E. grandis,* as both EgLBD15 and EgLBD11 do not contain the Pro residue.

**Figure 1:**
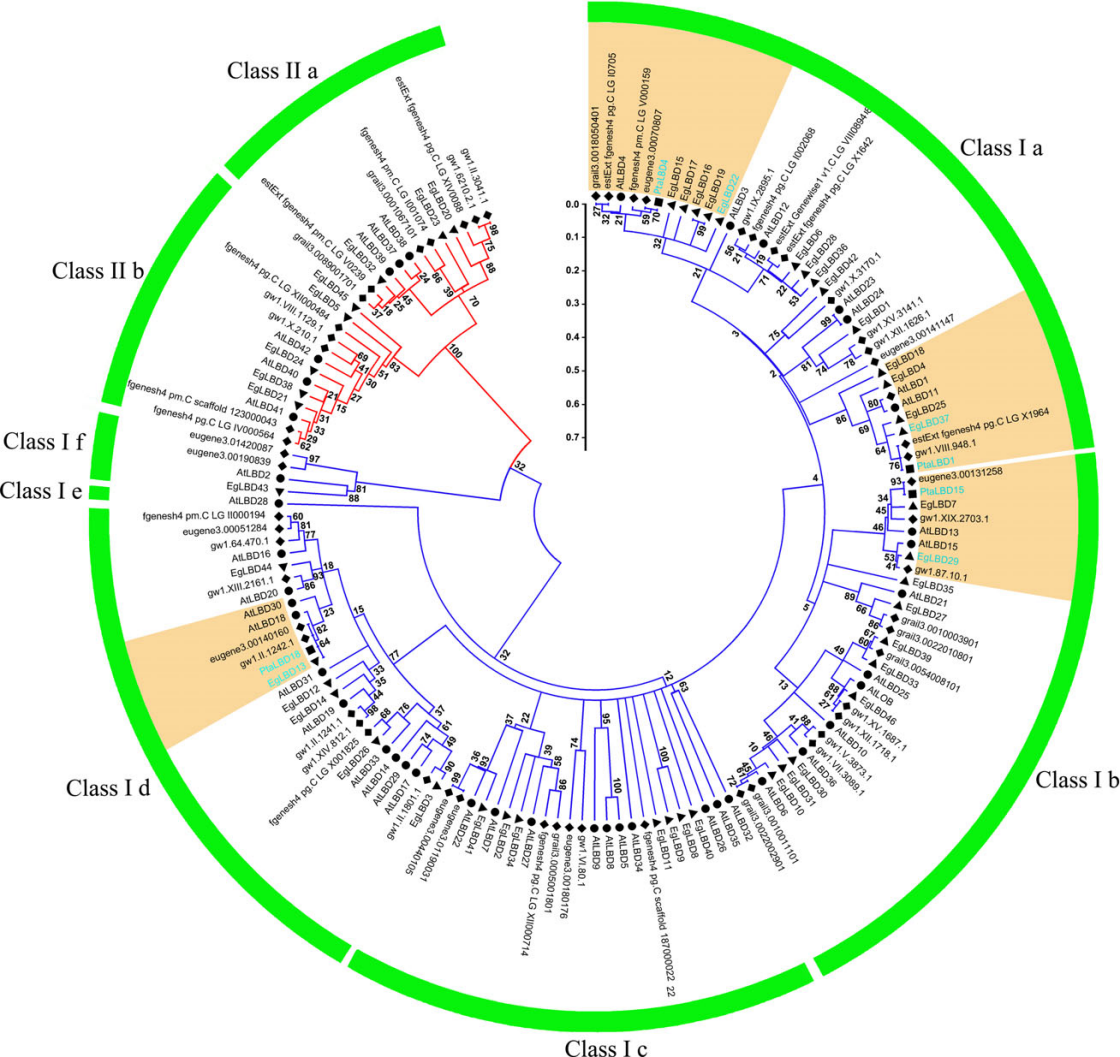
The phylogenetic relationship of LBD proteins in *Eucalyptus grandis*,* Arabidopsis thaliana*,* Populus trichocarpa* and *Populus tremula* × *Populus alba*. Triangle (▲), circle (●), rhombus (♦), square(■) represent *E. grandis*,* A. thaliana*,* P. trichocarpa* and *P*.* tremula* × *P. alba*, respectively. The blue branches represent class I, and the red branches represent class II. Each LBD subclass is indicated by a green arc (class I a‐f and class II a‐b);, the homologs of PtaLBD1, PtaLBD4, PtaLBD15, PtaLBD18 are in yellow shadow, PtaLBD1, PtaLBD4, PtaLBD15, PtaLBD18, EgLBD13, EgLBD22, EgLBD29 and EgLBD37 are shown in sky blue font. The numbers on the branches mean the reliability per cent of bootstraps value based on 1000 replication, the scale bar represents 0.1 substitutions per amino acid.

To investigate the evolutionary relationships of the *E. grandis* LBD proteins, sequences of the 46 EgLBDs, all known Arabidopsis LBD proteins and *Populus trichocarpa* LBD proteins including four *Populus tremula* × *Populus alba* LBD (PtaLBD) proteins implicated in secondary growth were used to construct a neighbour‐joining (NJ) phylogenetic tree. According to evolutionary relationships, 46 EgLBDs were classified into eight subgroups (subgroups a to f within class I and subgroups a and b within class II) (Figure 1). Among them, 14 EgLBDs belonged to class I a, 10 EgLBDs fell into class I b. Three (PtaLBD1, PtaLBD4, PtaLBD15) out of four LBD proteins (PtaLBD1, PtaLBD4, PtaLBD15 and PtaLBD18) associated with secondary growth that have been identified in *Populus tremula* × *Populus alba* (Yordanov and Busov, 2011) fell into these two subgroups, while PtaLBD18 belonged to a different subgroup (class I d). Interestingly, EgLBD22 and PtaLBD4, EgLBD37 and PtaLBD1, EgLBD29 and PtaLBD15, EgLBD13 and PtaLBD18 were clustered into the same clade (Figure 1). Therefore, we picked EgLBD22, EgLBD29 and EgLBD37 as close homologs of the poplar proteins for further functional analysis.

**Figure 2:**
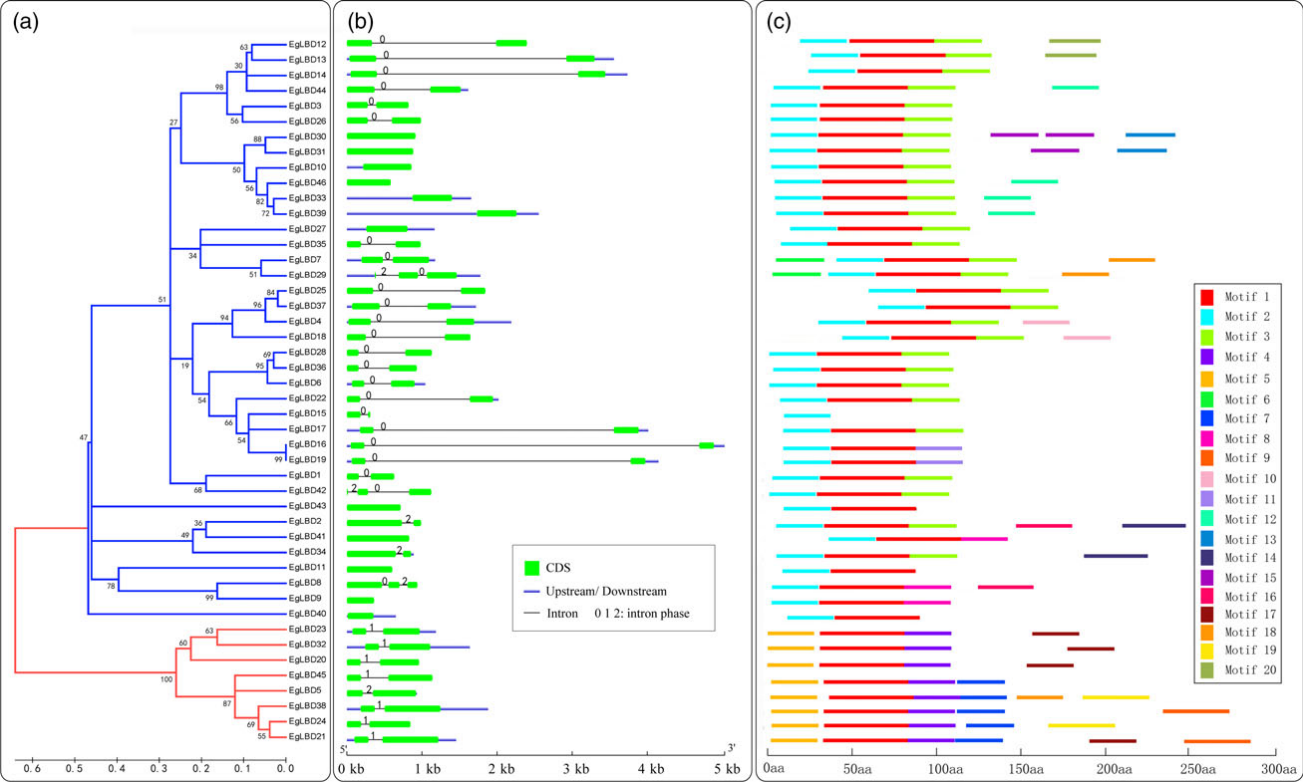
The phylogenetic relationship, gene structure and motif composition analysis of the *LBD* gene family in *Eucalyptus grandis*. (a) The amino acid sequences of the EgLBD proteins were aligned with Clustal X, and the phylogenetic tree was constructed using the neighbour‐joining method in MEGA5; the scale bar represents 0.1 substitutions per amino acid, and the branches of different classes were painted with different colours (the blue branches represents class I, the red branches represents class II). (b) Exon/intron structures of the *EgLBD* genes, the exons, introns and UTR are represented by the green boxes, black lines and blue boxes, respectively, and the scale bar represents 1 kb. (c) Conserved motif of EgLBD proteins, different motifs are represented by different coloured boxes with number 1–20, and the scale bar represents 50 aa (amino acid).

Structural features of EgLBD proteins were investigated as a function of their phylogeny. By analysing the structure of *EgLBD* genes, we revealed that closely related members had a similar exon/intron structure and gene length. The number of exons ranged from one to three, with 31 genes having two exons, 12 genes having one exon and only three genes (*EgLBD8*,* EgLBD29* and *EgLBD42*) having three exons (Figure 2a, b). To further understand the EgLBD functional regions, conserved motifs were predicted by MEME. Twenty individual motifs were identified (Figure 2c). Motif sequences were provided in Table S4. Our results showed that the length of motifs ranged from 30 to 70 amino acids and the number of motifs varied between 1 and 6 in each EgLBD protein. Conserved motif 1, motif 2 and motif 5 were most closely related to DUF260 (Domain of Unknown Function 260) (Table S4) based on CDART analysis. Notably, all EgLBDs contain motif 1 except EgLBD15, motif 2 was widespread in class I, motif 4 and 5 were widespread in class II, while other motifs were specific for a particular subgroup. These data suggest that the motifs in EgLBD proteins play critical roles in specific functions or have similar functionality.

The 46 *EgLBD* genes were unevenly distributed on the eleven chromosomes (Figure S2). Chromosome 10 contains the largest number with eight *EgLBD* genes, followed by chromosome 5 and chromosome 7 (seven genes per chromosome). Chromosome 1 and chromosome 4 contained the least, with only one *EgLBD* gene on them. Furthermore, we observed that distribution of each type of *LBD* gene was uneven, as three paralogous gene pairs (*EgLBD8/EgLBD9*,* EgLBD16/EgLBD19* and *EgLBD30/EgLBD31*) were located in the same chromosome, but the other ten paralogous gene pairs (*EgLBD1/EgLBD42*,* EgLBD2/EgLBD41*,* EgLBD3/EgLBD26*,* EgLBD7/EgLBD29*,* EgLBD12/EgLBD13*,* EgL BD23/EgLBD32*,* EgLBD24/EgLBD21*,* EgLBD25/EgLBD37*,* EgLBD28/EgLBD36*,* EgLBD33/EgLBD39*) were located on different chromosomes. In *EgLBD* family, we found that *EgLBD15*,* EgLBD16* and *EgLBD17* were tandem duplicated locus on chromosome 5. The percentage of tandemly duplicated genes within this gene family was very low and only reached 6.5% (3/46), compared to the average of 34% across the whole genome annotation (Myburg *et al*., 2014).

### Tissue‐specific expression of *EgLBD* genes and response to GA and IAA treatment

**Figure 3:**
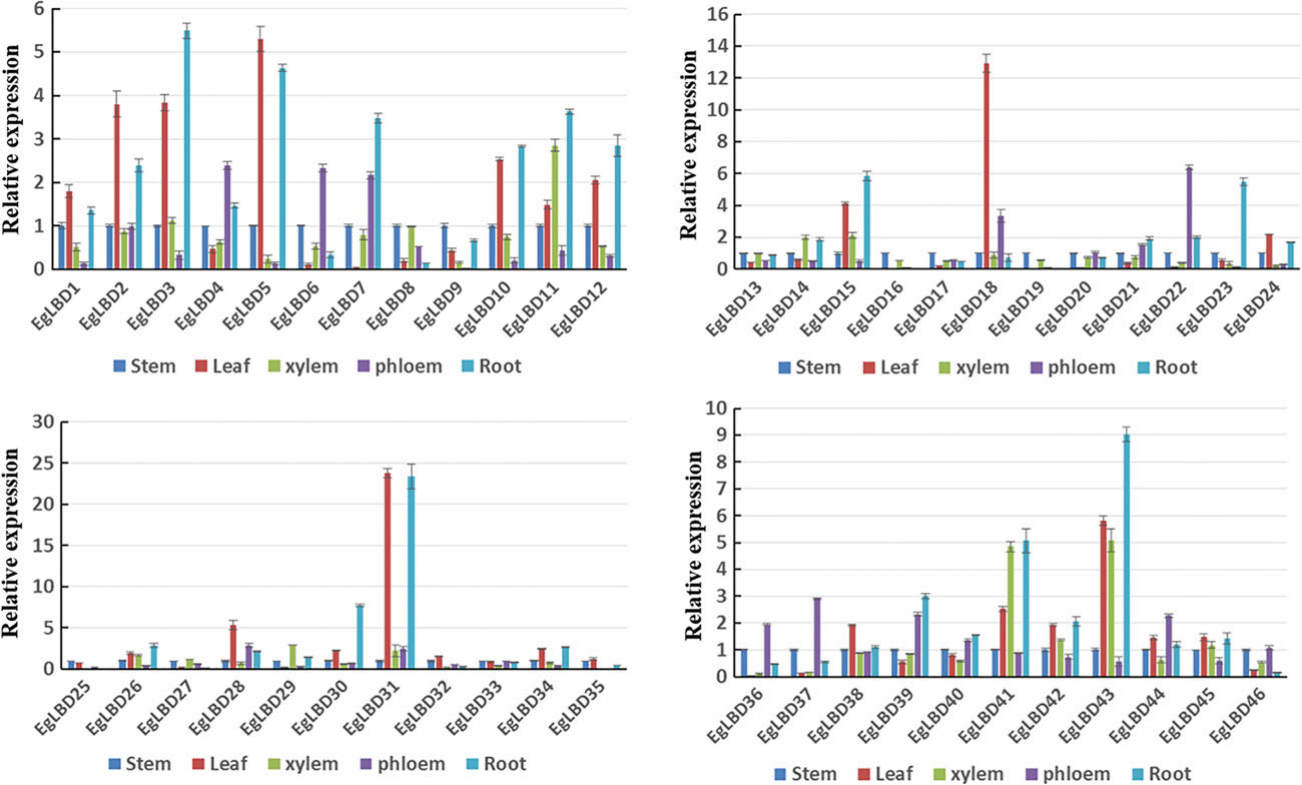
Expression patterns of the 46 *LBD* genes in root, stem, leaf, xylem and phloem of *Eucalyptus grandis* by qRT‐PCR analysis. Bars indicate standard deviations (SD) (*n *=* *3).

The expression profile of a *LBD* gene can reflect its biological function. To find candidate *EgLBD* genes involved in secondary cell wall development, we employed qRT‐PCR to detect the expression patterns of *EgLBD* genes in *E. grandis* root, stem, leaf, xylem and phloem. As shown in Figure 3, *EgLBD22* was preferentially expressed in the phloem and *EgLBD29* gene expression levels were highest in the xylem. Like *EgLBD22*, the expression of *EgLBD37* was also predominately expressed in phloem.

**Figure 4:**
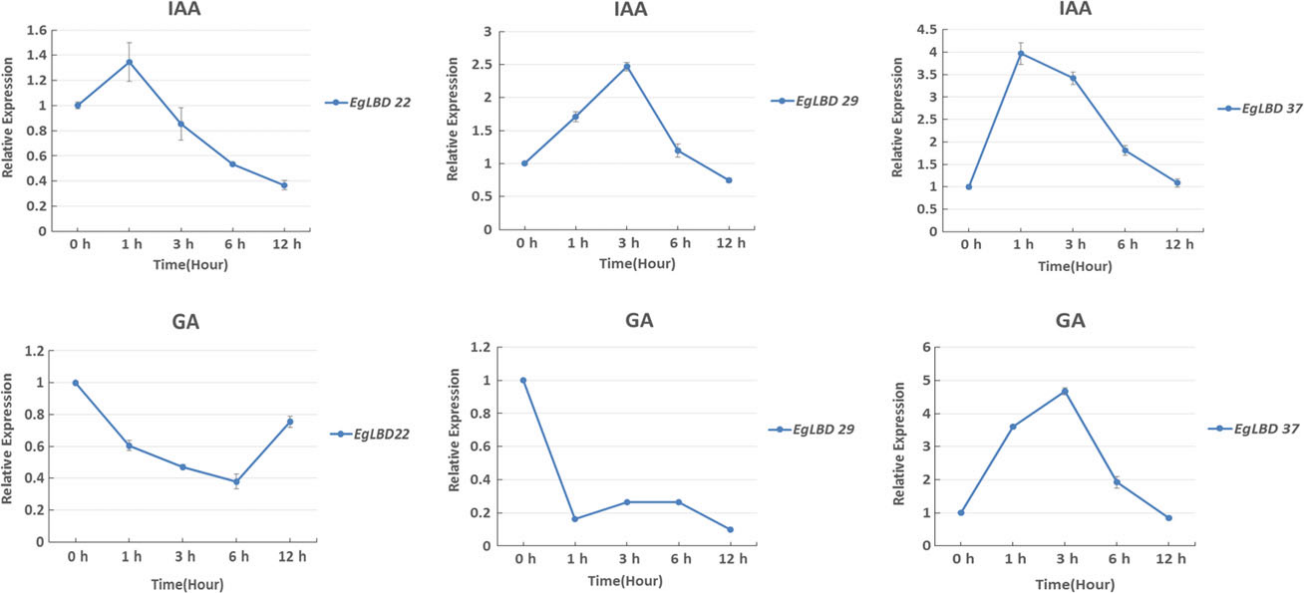
Expression analysis of *EgLBD22, EgLBD29* and *EgLBD37* genes under IAA and GA3 treatment by qRT‐PCR. Bars represent standard deviation for three replicates.

To assess the transcriptional responses of *EgLBD* genes to hormone treatments, the roots of 2‐month‐old *E. grandis* seedlings were treated with 150 μm of IAA and 150 μm of GA_3_, respectively. The expression levels of three *EgLBD* genes (*EgLBD22, EgLBD29* and *EgLBD37*) were analysed by qRT‐PCR with stem‐derived RNA after the treatment of IAA and GA_3_ for 0 h, 1 h, 3 h, 6 h and 12 h (Figure 4). The results showed that *EgLBD29* (2.5‐fold) and *EgLBD37* (fourfold) were highly up‐regulated by IAA treatment with *EgLBD22* displaying only a minor up‐regulation after only 1 h (1.4‐fold). All IAA gene expression responses peaked within 1‐3 h and then declined, with the expression levels of *EgLBD22* declining to 0.4‐fold after 12 h. The GA_3_ response of *EgLBD37* showed a 4.7‐fold increase after 3 h and then declined back to normal after 12 h. However, *EgLBD22* and *EgLBD29* expression dropped to 0.4 and 0.2, respectively, at 6 h and 1 h under GA_3_ treatment. *EgLBD29* gene expression remained low throughout the 12‐h period, while the expression of *EgLBD22* increased back to 0.8‐fold after 12 h.

### Subcellular localization and possible functional partners of EgLBD proteins

We predicted the possible localization of EgLBD proteins using the protein subcellular localization prediction tool Plant‐mPLoc ( http://www.csbio.sjtu.edu.cn/bioinf/plant-multi/). The results showed that all EgLBD proteins were predicted to be localized to the nucleus (Table S3). To validate the predicted localizations, EgLBD22, EgLBD29 and EgLBD37 were transiently expressed in tobacco. The three EgLBD proteins were successfully expressed as fluorescent protein fusions. As shown in Figure S3, the fluorescent signals from fusion proteins LBD22‐GFP, LBD29‐GFP and LBD37‐GFP were all only observed in the nuclei of transformed tobacco leaf cells, while the fluorescence of protein encoded *35S::GFP* could be observed everywhere in the cell including the membrane, cytoplasm and nucleus. Therefore, the prediction for subcellular location of EgLBD22, EgLBD29 and EgLBD37 proteins was confirmed by our transient expression experiment. To find out the possible functional protein association networks, protein–protein interactions were predicted using STRING ( http://string-db.org/). The results of protein–protein interaction prediction for functional protein association networks revealed that EgLBD22, EgLBD29 and EgLBD37 had 4, 10, 2 possible functional partners, respectively, all of them consisting of different proteins. The partner with the highest score for EgLBD22, EgLBD29 and EgLBD37 was EXPA14 (EXPANSIN A14), F3F9.16 (general regulatory factor 2) and DOF (DSB formation protein), respectively (Table S5‐S7).

### Effects of key *EgLBDs* on secondary growth when overexpressed

**Figure 5:**
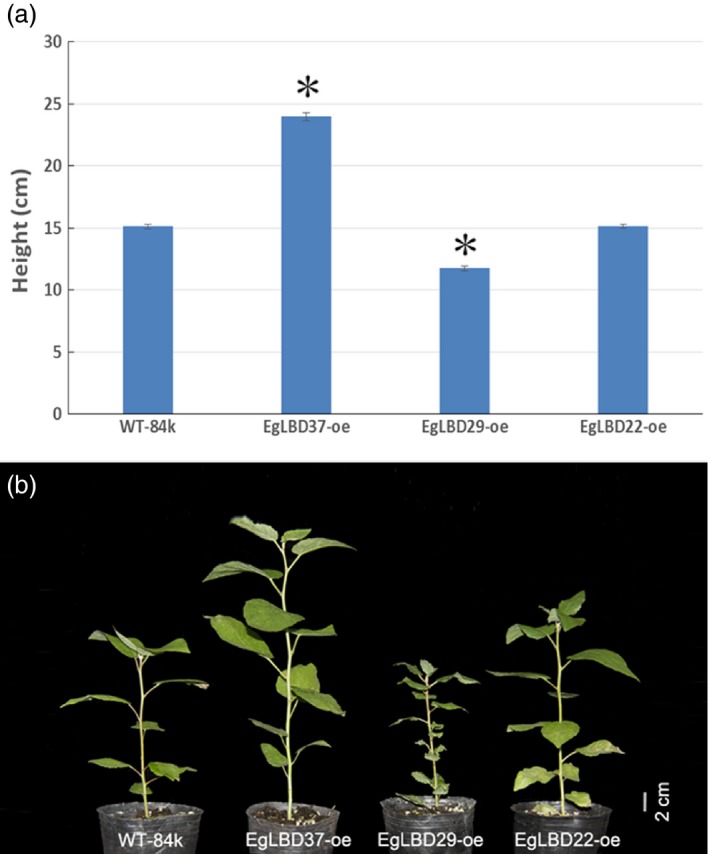
Plant height of *EgLBD22‐oe*,* EgLBD29‐oe* and *EgLBD37‐oe* and wild‐type hybrid poplar (*Populus glandulossa* × *Populus alba*) trees. (a) Average height data for ten‐week‐old wild‐type 84k (*Populus glandulossa* × *Populus alba*), *EgLBD22‐oe*,* EgLBD29‐oe* and *EgLBD37‐oe* plants. Bars indicate standard errors (SE), and asterisks indicate significant differences relative to control plants (WT‐84k) with * denoting *P* < 0.01. Values are reported as means ± SE (*n* = 5). (b) Ten‐week‐old wild‐type 84k, *EgLBD22‐oe*,* EgLBD29‐oe* and *EgLBD37‐oe* plants.

**Figure 6:**
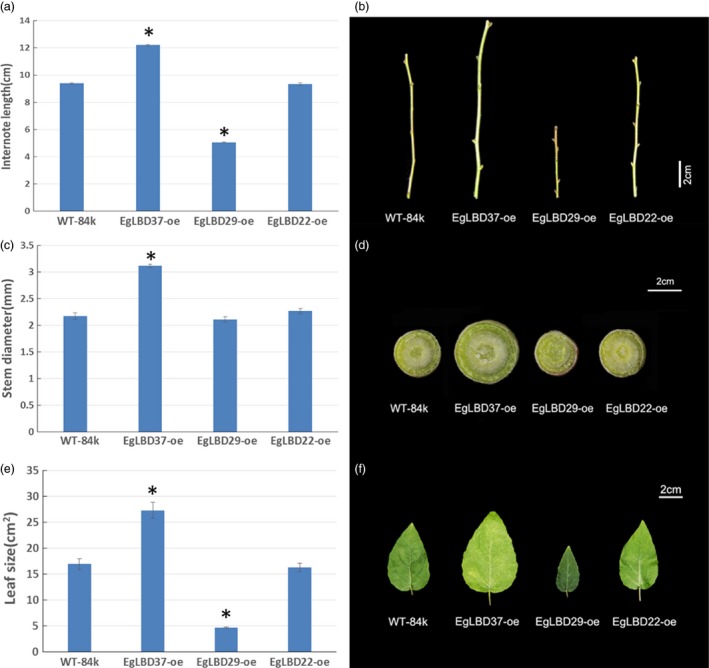
Internode length, stem diameter and leaf size of *EgLBD22‐oe*,* EgLBD29‐oe* and *EgLBD37‐oe* and wild‐type hybrid poplar (*Populus glandulossa* × *Populus alba*) trees. (a) Total internode length from 3rd to 8th nodes for ten‐week‐old wild‐type 84k, *EgLBD22‐oe*,* EgLBD29‐oe* and *EgLBD37‐oe* plants. (b) Representative images of the internode from 3rd to 8th nodes of ten‐week‐old wild‐type 84k, *EgLBD22‐oe*,* EgLBD29‐oe* and *EgLBD37‐oe* plants. (c) Average stem diameter data for ten‐week‐old wild‐type 84k, *EgLBD22‐oe*,* EgLBD29‐oe* and *EgLBD37‐oe* plants. (d) Representative stem cross section at the base of ten‐week‐old wild‐type 84k, *EgLBD22‐oe*,* EgLBD29‐oe* and *EgLBD37‐oe* plants. (e) Average area of the 6th leaf from the top of ten‐week‐old wild‐type 84k, *EgLBD22‐oe*,* EgLBD29‐oe* and *EgLBD37‐oe* plants. (f) The 6th leave from the top of ten‐week‐old wild‐type 84k, *EgLBD22‐oe*,* EgLBD29‐oe* and *EgLBD37‐oe* plants. Bars indicate standard error (SE), and asterisks indicate significant differences relative to control plants (WT‐84k) with * denoting *P* < 0.01. Values are reported as means ± SE (*n* = 5).

To characterize the functions of key *EgLBDs*, multiple transgenic lines of hybrid poplar (*Populus alba* × *Populus glandulosa*) clone 84k carrying the constructs *35S::LBD22*,* 35S::LBD29* and *35S::LBD37* were generated, validated for the presence and expression of the transgenes (Figures S4 and S5). The most striking phenotype of *EgLBD37‐oe* plants is that all the transgenic lines were much taller (up to 59%) than wild‐type 84 k trees (Figure 5) and showed a great increase in internode length (up to 30%) (Figure 6a, b), diameter of stem (up to 44%) (Figure 6c, d) and leaf size (up to 61%) (Figure 6e, f). In contrast, the most obvious phenotype of *EgLBD29‐oe* plants was that the height of all the transgenic lines was much smaller (less than 22%) than wild‐type 84k trees (Figure 5) and showed a dramatic reduction in internode length (less than 46%) (Figure 6a, b) and leaf size (less than 72%) (Figure 6e, f), while *35S::EgLBD22* did not exhibit any obvious phenotypes (Figure 5, Figure 6).

**Figure 7:**
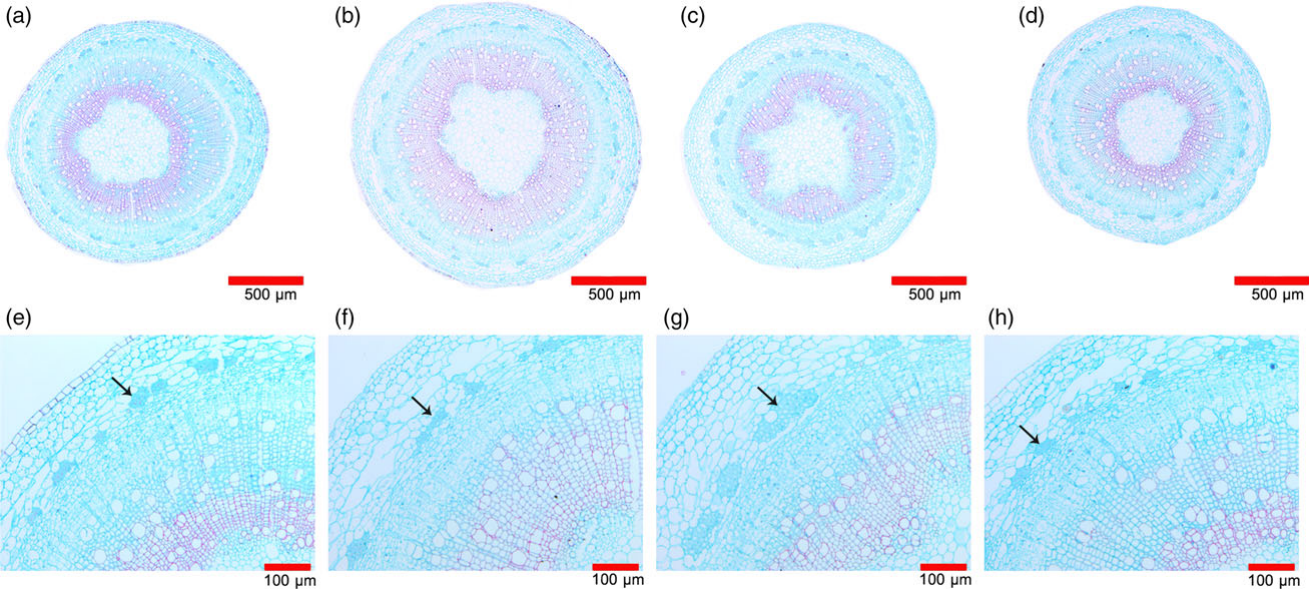
Stem anatomy of *EgLBD22‐oe*,* EgLBD29‐oe* and *EgLBD37‐oe* and wild‐type hybrid poplar (*Populus glandulossa* × *Populus alba*) trees. Fixed stem sections were stained with safranin and fast green (Gefan biotechnology Co. Ltd., Shanghai). (a, e) Stem structure of ten‐week‐old wild‐type 84k plant. (b, f) Stem structure of ten‐week‐old *EgLBD37‐oe* plant. (c, g) Stem structure of ten‐week‐old *EgLBD29‐oe* plant. (d, h) Stem structure of ten‐week‐old *EgLBD22‐oe* plant. In *EgLBD37‐oe* plant, the overall width of cortex region and the lignified component was significantly increased (b, f). Increased phloem fibre production in the *EgLBD29‐oe* plant was observed (arrow in g) when compared with wild‐type 84k (arrow in e), *EgLBD37‐oe* (arrow in f) and *EgLBD22‐oe* plant (arrow in h).

To evaluate the impact of *EgLBD22*,* EgLBD29* and *EgLBD37* on secondary growth, we sectioned the stems of the 10th node that undergo secondary growth from the control plant WT‐84k and *EgLBD22‐oe*,* EgLBD29‐oe* and *EgLBD37‐oe* transgenics. An obvious difference in stem anatomy could be observed under light microscopy between WT‐84k, *EgLBD37‐oe* and *EgLBD29‐oe* transgenic plants (Figure 7a–c). In *EgLBD37‐oe* plants, the overall width of cortex region and the lignified component of the secondary xylem were significantly increased (Figure 7b, f). There was also an increase in the thickness of the cortex region and the lignified component of the secondary xylem in *EgLBD29‐oe* (Figure 7c, g). The most profound change in stem anatomy for *EgLBD29‐oe* transgenics was that all the transgenic lines produced more phloem fibres (Figure 7c, g) when compared with WT‐84k (Figure 7a, e), *EgLBD22‐oe* (Figure 7d, h) and *EgLBD37‐oe* transgenics (Figure 7b, f). For *EgLBD22*‐*oe* transgenics, no significant phenotypic changes could be detected when it was compared with that of the control WT‐84k (Figures 5, 6, 7d, h).

### Transcriptome changes in *EgLBD22‐oe*,* EgLBD29‐oe*,* EgLBD37‐oe* plants

To gain further insight into the biological functions of *EgLBD22*,* EgLBD29* and *EgLBD37*, we carried out RNA‐seq analysis of *LBD22‐oe, EgLBD29‐oe* and *EgLBD37‐oe* hybrid poplar (*Populus alba* × *Populus glandulosa*) plants. Compared with WT‐84k plant, there were 299, 421 and 118 unique sequences showing at least onefold changes in *EgLBD22‐oe*,* EgLBD29‐oe* and *EgLBD37‐oe* plants, respectively, (Tables S8–S10). In *EgLBD22‐oe* plant, the *expansin* gene c60809_g2 decreased 1.3‐fold and an *auxin efflux carrier component 6* gene decreased 1.5‐fold, but a class‐I *KNOX* gene (c70392_g3) was shown to be up‐regulated to 1.3‐fold (Table S8). In *EgLBD29‐oe* plant, the expression of ethylene‐responsive transcription factor *ERF023*‐like gene increased 3.8‐fold and an ethylene‐responsive element‐binding family protein‐encoding gene increased 1.8‐fold, while the *expansin* gene c60809_g2 and *expansin* gene c60754_g1 was reduced 1.1‐ and 1.2‐fold, respectively (Table S9). Consistent with our expectation, a MYB family transcription factor *APL*‐like gene (c62428_g1) was observed to be up‐regulated to 1.8‐fold in *EgLBD29‐oe* plant (Table S9). However, there were no alterations in the expression level of *APL*‐like gene in *EgLBD22‐oe* plant and *EgLBD37‐oe* plant when compared with that of the WT‐84k plant (Tables S8 and S10). Notably, *Populus trichocarpa gibberellin‐regulated protein 5* was up‐regulated to 4.6‐fold and *expansin‐B3* was up‐regulated to 1.5‐fold in *EgLBD37‐oe* plants (Table S10); however, no changes in the expression of class‐I *KNOX* genes were detected in *EgLBD29‐oe* plant and *EgLBD37‐oe* plant as compared to that of WT‐84k plant (Tables S9 and S10).

**Figure 8:**
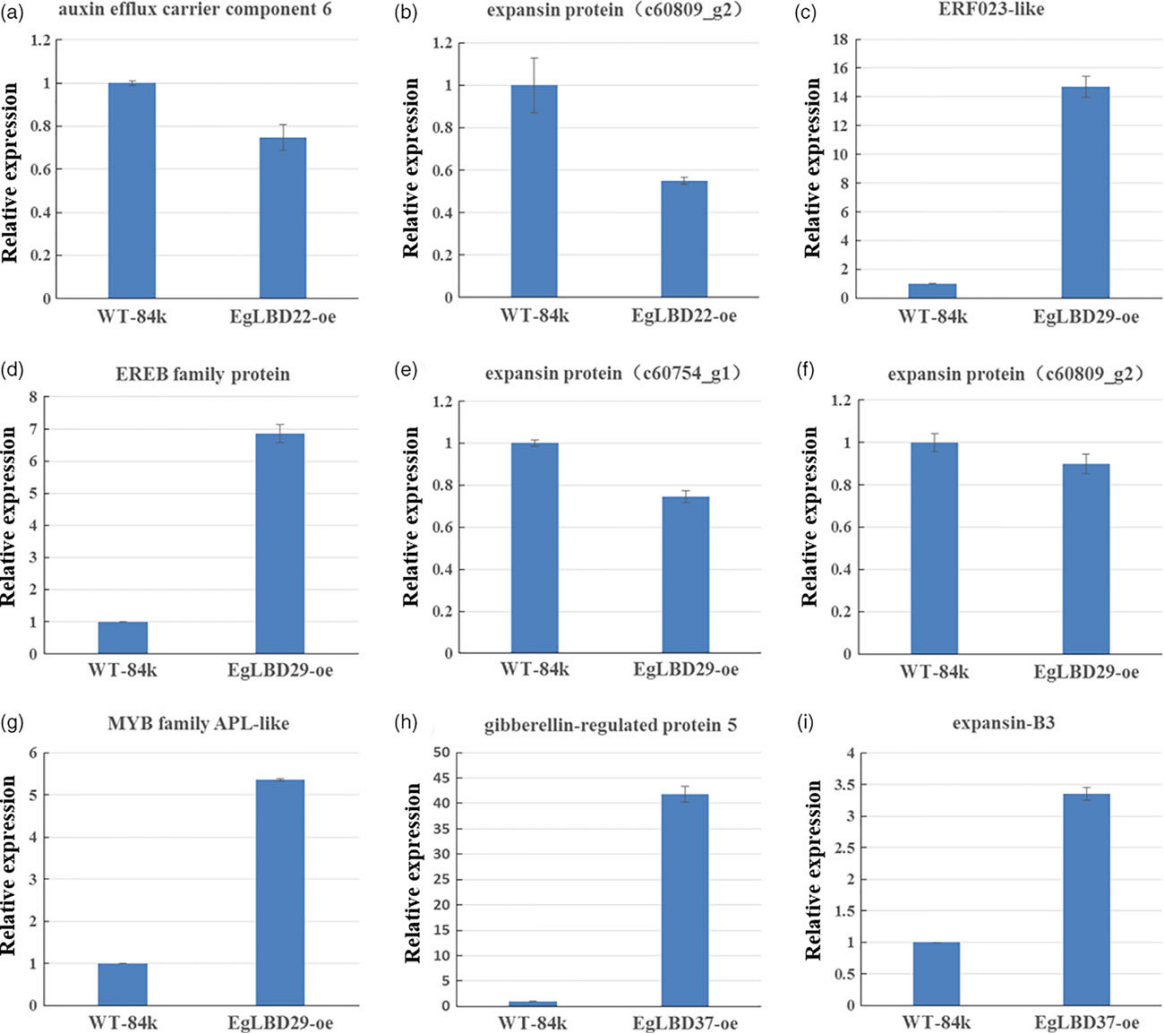
Expression analysis of selected genes in *EgLBD22‐oe*,* EgLBD29‐oe* and *EgLBD37‐oe* plants by qRT‐PCR. (a) Comparison of the expression level of auxin efflux carrier component 6 encoding gene between WT‐84k and *EgLBD22‐oe* plants. (b) Comparison of the expression level of expansin protein‐encoding gene (c60809_g2) between WT‐84k and *EgLBD22‐oe* plants. (c) Comparison of the expression level of ethylene‐responsive transcription factor ERF023‐like protein‐encoding gene between WT‐84k and *EgLBD29‐oe* plants. (d) Comparison of the expression level of ethylene‐responsive element‐binding (EREB) family protein‐encoding gene between WT‐84k and *EgLBD29‐oe* plants. (e) Comparison of the expression level of expansin protein‐encoding gene (c60754_g1) between WT‐84k and *EgLBD29‐oe* plants. (f) Comparison of the expression level of expansin protein‐encoding gene (c60809_g2) between WT‐84k and *EgLBD29‐oe* plants. (g) Comparison of the expression level of *APL*‐like gene (c62428_g1) between WT‐84k and *EgLBD29‐oe* plants. (h) Comparison of the expression level of gibberellin‐regulated protein 5 encoding gene between WT‐84k and *EgLBD37‐oe* plants. (i) Comparison of the expression level of expansin‐B3 protein‐encoding gene between WT‐84k and *EgLBD37‐oe* plants. Bars represent standard deviation for three replicates. All expression estimates were normalized to the expression of an actin loading control gene.

To verify the gene expression results obtained from RNA‐seq experiments, we chose eight representative genes (*gibberellin‐regulated protein 5*,* expansin‐B3*, ethylene‐responsive transcription factor *ERF023*‐like gene, ethylene‐responsive element‐binding family protein‐encoding gene c59103_g1, *expansin* gene c60809_g2, *expansin* gene c60754_g1, *auxin efflux carrier component 6* gene and *APL*‐like gene c62428_g1) (Table S11) and monitored their expression levels using qRT‐PCR method. The results showed that these genes were significantly up‐regulated or down‐regulated in the *EgLBD22‐oe*,* EgLBD29‐oe* and *EgLBD37‐oe* plants as compared to the control WT‐84k (Figure 8), which is in good agreement with the results obtained by our RNA‐seq analysis of *EgLBD22‐oe*,* EgLBD29‐oe* and *EgLBD37‐oe* plants.

## Discussion

*LBD* genes exist ubiquitously in plants and are important regulators of plant‐specific processes (Majer and Hochholdinger, 2011; Xu *et al*., 2016). In this study, forty‐six *EgLBDs* were identified from the *E. grandis* genome sequences. The *LBD* gene family in *E. grandis* is similar to the estimates for other reported plant species, such as the forty‐three *LBD* genes in Arabidopsis and thirty‐five in rice (Shuai *et al*., 2002; Yang *et al*., 2006). Given that tandem duplications were reported to be 34% on average in the *E. grandis* genome (Myburg *et al*., 2014) and tandem duplications in the *EgLBD* family was only 6.5% (3/46) in this investigation, we conclude that the evolution of this family in *E. grandis* was not significantly affected by tandem duplications.

Structural analysis is a powerful method to mine valuable information concerning duplication events and phylogenetic relationships of genes within a gene family. In this study, we observed that *LBD* genes in *E. grandis* had simple gene structures and that most *EgLBD* members within the same subgroup had a similar exon/intron structure and gene length. This is similar to the *LBD* genes in *Arabidopsis thaliana*, rice and apple (Shuai *et al*., 2002; Wang *et al*., 2013b; Yang *et al*., 2006). Therefore, we infer that the structures of *LBD* genes are relatively conserved in different angiosperms. Through analysis of EgLBD proteins within each subgroup, we demonstrated that they generally possessed similar protein motifs. Among the motifs, the designation of motifs 1, 2 and 5 was DUF260 (domain of unknown function 260) (Table S4). EgLBD15 only had motif 2, while all the other class I EgLBD proteins had motif 1 and motif 2, and all the class II EgLBD proteins had motif 1 and motif 5. Because DUF260 motif contains the conserved DNA binding motif (CX_2_CX_6_CX_3_C zinc finger‐like motif), we believe that this protein motif is likely to play a role in transcriptional regulation of their target genes.

The mechanisms by which *LBD* genes control root and leaf development have been elucidated. Hay *et al*. (2006) demonstrated that auxin and ASYMMETRIC LEAVES1 (AS1) converged to repress expression of the *KNOTTED1*‐like homeobox (*KNOX*) gene *BREVIPEDICELLUS* (*BP*) and thus promote leaf development. Regulation of lateral root development by *AtLBD16*,* AtLBD28, AtLBD29* and *AtLBD33* was found to be associated with the auxin signal transduction pathway, and these genes were directly regulated by AtARF7 and AtARF19 through auxin responsive elements (Lee *et al*., 2009; Okushima *et al*., 2007). During lateral root initiation, AtLBD18 and AtLBD33 had been proven to activate plant cell division through transcriptional regulation of Arabidopsis *E2Fa* (Berckmans *et al*., 2011) or by directly binding to the promoter of *EXPANSIN14* (Lee *et al*., 2013). Arabidopsis *JAGGED LATERAL ORGANS* (a member of the *LBD* gene family) acts with *ASYMMETRIC LEAVES2* to coordinate *KNOTTED1*‐like homeobox (*KNOX*) and *PIN‐FORMED1* (*PIN1*) expression during shoot and root development in Arabidopsis (Rast and Simon, 2012). However, there is still limited knowledge about the mechanisms by which *LBD* genes control secondary growth. Yordanov *et al*. (2010) reported that two key class I *KNOTTED1‐*like homeobox genes (*ARBORKNOX1* and *ARBORKNOX2*) that promote meristem identity in the cambium were down‐regulated, while *APL* was up‐regulated in *PtaLBD1‐oe* plants showing enhanced woody growth and remarkable changes in bark texture. Based on these findings, they proposed a mechanistic model of *LBD* regulatory roles in secondary woody growth (Yordanov and Busov, 2011). In this model, *PtaLBD1* and *PtaLBD4* are expressed at the cambium/phloem boundary and regulate secondary phloem development by restraining the expression of meristem identity genes (*ARBORKNOX1* and *ARBORKNOX2*) in the cambium zone and promote phloem development through activation of *APL* and likely other unknown set of genes. *PtaLBD18* and *PtaLBD15* are expressed at the cambium/xylem border and also restrict the expression of meristem identity genes to the cambium zone while at the same time promote xylem development through activation of other unknown genes. *PtaLBDs* play antagonistic roles with meristem maintenance genes to maintain meristem identity in cambium and/or promote xylem/phloem/ray cell tissue differentiation. Although this model seems plausible, the molecular mechanisms underlying secondary growth remain to be further elucidated.

Members of *LBD* genes in Arabidopsis, rice and other species have been found to respond to the treatments of cytokinin, gibberellic acid, auxin, brassinosteroid and abscisic acid (Bell *et al*., 2012; Berckmans *et al*., 2011; Naito *et al*., 2007; Wang *et al*., 2013b Zentella *et al*., 2007). Using qRT‐PCR method, we have demonstrated that expression of some *EgLBD* genes also responded to auxin and gibberellin (Figure 4). The expression levels of *EgLBD22*,* EgLBD29* and *EgLBD37* were all transiently up‐regulated following exogenous auxin treatment (Figure 4). Interestingly, *EgLBD37* was up‐regulated, whereas *EgLBD22* and *EgLBD29* were down‐regulated under exogenous gibberellic acid treatment (Figure 4), suggesting that the *EgLBD22/29* and *EgLBD37* genes may differentially regulate GA‐mediated growth and developmental processes in *E. grandi*s. *Gibberellic acid‐stimulated Arabidopsis6* (*AtGASA6*) promoted cell elongation during the germination of Arabidopsis seeds (Zhong *et al*., 2015). In this study, we demonstrated that *gibberellin‐regulated protein5* in hybrid poplar was up‐regulated to 4.6‐fold in *EgLBD37‐oe* plants (Table S10). Given that the link between the transcriptional response of *EgLBD37* to GA treatment and dramatic increase in secondary growth and the connection between the expression level of *gibberellin‐regulated protein5* and internode length of *EgLBD37‐oe* plants, it is reasonable for us to conclude that *EgLBD37* enhances secondary growth and plant stem elongation through GA‐mediated signalling pathway. Moreover, we found that *expansin‐B3* was up‐regulated in *EgLBD37‐oe* plants (Table S10), leading us to conclude that, like AtLBD18, EgLBD37 promotes cell expansion during secondary growth via activating the expression of *expansin* genes.

In our experiment, an ethylene‐responsive transcription factor ERF115 was predicted to be the functional partner of EgLBD29 (Table S6). Interestingly, we found that the expression of ethylene‐responsive transcription factor *ERF023*‐like gene increased 3.8‐fold and the expression of an ethylene‐responsive element‐binding family protein‐encoding gene (*EREBP*) increased 1.8‐fold in *EgLBD29‐oe* plants, which showed a significant broader phloem fibres region (Table S9, Figure 7g). Recently, a similar plant morphology to *EgLBD29‐oe* lines was observed in *pLMX5::ERF139‐*overexpressing hybrid aspen (*Populus tremula × Populus tremuloides*) which showed a dwarf phenotype with altered wood development (Vahala *et al*., 2013). It has already been well documented that ethylene can enhance plant fibre development (Shi *et al*., 2006). All of these evidences suggest that there is an ethylene‐mediated signalling pathway for the function of *EgLBD29* in control of secondary growth, especially phloem fibre development. The *expansin* gene c60809_g2 and *expansin* gene c60754_g1 was reduced 1.1‐ and 1.2‐fold in *EgLBD29‐oe* plants (Table S9), while *Expansin‐B3* was up‐regulated to 1.5‐fold in *EgLBD37‐oe* plants (Table S10). Because the phenotype of *EgLBD29‐oe* plants contrasted to that of *EgLBD37‐oe* plants in terms of height, leaf size and stem diameter (Figures 5 and 6), it is likely that EgLBD29 and EgLBD37 exert opposite influences on cell growth and cell wall expansion during secondary growth through their opposite roles in control of the expression of *expansin* genes.

We found that overexpression of *EgLBD37* not only significantly increased diameter of stem (Figure 6c, d), but also dramatically increased plant height (Figure 5), internode length (Figure 6a, b) and leaf size (Figure 6e, f). Like *EgLBD37*, overexpression of *PtaLBD1* increased stem diameter of transgenic plants via promoting secondary growth. However, overexpression of *PtaLBD1* did not increase plant height, internode length and leaf size (Yordanov *et al*., 2010). Why did *EgLBD37‐oe* affect the cortex thickness and xylem formation but not phloem development? One plausible explanation is that EgLBD37 is not the functional ortholog of PtaLBD1 (for example, it could be EgLBD25 according to Figure 1). Another explanation could be that there is a functional divergence in terms of target genes between EgLBD37 and PtaLBD1. In addition, we found that *EgLBD29‐oe* plants have reduced plant height (Figure 5), shorter internode length (Figure 6a, b), thinner stem diameter (Figure 6c, d) and smaller leaf size (Figure 6e, f), which contrasts to the phenotype of *EgLBD37‐oe* plants, implying that *EgLBD29* and *EgLBD37* may have an antagonistic function. The potential application of *EgLBD37* would be to increase timber production and *EgLBD29* would be to improve pulp yield because of high‐quality fibres that may come from *EgLBD29‐oe* transgenic trees.

In conclusion, we have described the evolutionary relationship of the *LBD* gene family in *E. grandi*s and functionally characterized three *EgLBD* genes, two of which showed significant effects on secondary growth. Our results will facilitate efforts to gain a deeper understanding of the structure‐function relationships of these genes and may enable novel breeding techniques to improve wood formation or fibre production in trees.

## Experimental procedures

### Plant materials and growth conditions

One‐year‐old *E. grandis* trees were planted in the greenhouse of Chinese Academy of Forestry under controlled conditions with a relative humidity of 50% at 25 °C. For detecting the expression level in different tissues, roots, stems, leaf, xylem and phloem of *E. grandis* were sampled in the spring of 2015. For phloem collection, a bark window approx. 30 × 50 mm was removed from the stem using a hammer and chisel. Xylem tissue was collected by scraping cells from the exposed wood with chisel. For determination of IAA (indol‐3‐acetic acid) and GA_3_ (gibberellic acid) response, roots of two‐month‐old seedlings (25–30 cm in height) were treated with 150 μm IAA (Sigma‐Aldrich China, Shanghai) or 150 μm GA_3_ (Biotopped, Beijing) solution for 0, 1, 3, 6 and 12 h, respectively. The first time point (0 h) served as a control. After IAA and GA_3_ solution treatment, stems were harvested from seedlings. Three biological replicates were performed for each sample. All the samples were immediately frozen in liquid nitrogen and stored at −80 °C freezer before total RNA isolation.

### Identification of *LBD* genes in *E. grandis*

The *E. grandis* genome sequence version 2.0 was downloaded from the Phytozome database ( http://www.phytozome.net/) (Goodstein *et al*., 2012) and used to construct a local BLAST database. All known *LBD* gene sequences of Arabidopsis, poplar and rice were downloaded from the GenBank ( https://www.ncbi.nlm.nih.gov/genbank/) of NCBI (National Center for Biotechnology Information). These sequences were used as query to perform local BLAST searches against the *E. grandis* genome with e‐value cut‐off set to 10^−5^. All candidate LBD protein sequences were examined by the domain analysis programs SMART (Simple Modular Architecture Research Tool) ( http://smart.embl-heidelberg.de/) (Letunic *et al*., 2015) and Pfam ( http://pfam.xfam.org/) (Punta *et al*., 2012) with the default parameters. We then analysed the *E. grandis* candidate LBD protein sequence domain using a hidden Markov model (HMM) as described by Wu *et al*. (2002).

### Bioinformatic analysis and phylogenetic analysis

ExPASy ( http://web.expasy.org/compute_pi/) was used to predict the pI and molecular weight (Artimo *et al*., 2012). All LBD proteins sequences from *A. thaliana, E. grandis*,* Populus trichocarpa* and four *Populus tremula* × *Populus alba* (Pta) LBD proteins were aligned using Clustal X (Thompson *et al*., 1997). Using MEGA5 software, the phylogenetic tree of full‐length sequences was constructed by the neighbour‐joining method with bootstrap to be 1000 (Tamura *et al*., 2011).

### Gene structure, conserved motif analyses and chromosomal location

*EgLBD* genes exon/intron structure was identified with Gene Structure Display Server 2.0 (GSDS, http://gsds.cbi.pku.edu.cn/) (Hu *et al*., 2015). Conserved motifs of the proteins were analysed using the Multiple Em for Motif Elucidation (MEME) program ( http://meme-suite.org/index.html) (Bailey and Elkan, 1994). MEME was used by setting repetitions to any number, the number of motifs to 20 and optimum motif width to 30–70. Functional designation of the motifs was performed to get valid domain hits for architecture search with CDART (Conserved Domain Architecture Retrieval Tool) reported by Geer *et al*. (2002), which can be accessed at https://www.ncbi.nlm.nih.gov/Structure/lexington/lexington.cgi. The chromosomal locations were retrieved from the genome data downloaded from the Phytozome database (Goodstein *et al*., 2012) and mapped to the chromosomes using MG2C (Map Gene2 Chromosome v2, http://mg2c.iask.in/mg2c_v2.0/).

### RNA extraction, cDNA synthesis and gene expression profiling

Total RNA was isolated with RNA extraction kit (TIANGEN, Beijing, China) from *E. grandis* samples including root, stem, leaf, xylem and phloem. The purity and quality of RNA was checked by NanoDrop8000 (Thermo Fisher Scientific, Waltham, MA, USA) and analysed by gel electrophoresis. One microgram of total RNA was reverse‐transcribed using the Prime‐Script RT reagent kit (Takara, China). qRT‐PCR was conducted with SYBR Premix EX Taq II (Takara, China). *PP2A‐3* (protein phosphatase 2A subunit A3) gene was used as internal reference, and each reaction was conducted in triplicate. The stem expression values were set to 1. Relative gene expression was calculated according to the delta–delta Ct method of the system. The primers used in qRT‐PCR analysis for tissue‐specific expression of *EgLBD* genes are listed in Table S1.

### Subcellular localization and protein–protein interaction prediction

Protein subcellular localization was predicted using Plant‐mPLoc ( http://www.csbio.sjtu.edu.cn/bioinf/plant-multi/). To validate subcellular localization, the full‐length coding sequences (without the stop codon) of *EgLBD22*,* EgLBD29* and *EgLBD37* were amplified from RNA of *E. grandis* stem by RT‐PCR. The PCR product of *EgLBD37* was digested with *Nco*I and *Spe*I and directionally ligated into vector pCAMBIA1302 to construct the *EgLBD37‐GFP* fusion gene driven by a CaMV35S promoter (Niwa, 2003). The PCR products of *EgLBD22* and *EgLBD29* were ligated to vector pCAMBIA1302, respectively, using Seamless Assembly Cloning Kit (CloneSmater, Beijing, China). The pCAMBIA1302‐GFP was used as the positive control. Transient expression in lower leaf epidermal cells of *Nicotiana tabacum* L. was performed as described by Zheng *et al*. (2005). The transient expression of the EgLBD22/29/37‐GFP fusion proteins was observed under Ultra‐VIEW VoX 3D Live Cell Imaging System Spinning Disk confocal laser scanning microscope (PerkinElmer, Waltham, MA, USA). All the primers used in subcellular localization are listed in Table S1. Protein–protein interaction prediction was performed using the online tool of STRING ( http://string-db.org/).

### Plant transformation

Plantlets of hybrid poplar (*Populus alba* × *Populus glandulosa*) clone 84k were grown at 23–25 °C under a 16/8 h day/night cycle with a light intensity of 50 μm/m^2^/s provided by cool white fluorescent tubes. The full‐length coding sequences of *EgLBD22*,* EgLBD29* and *EgLBD37* were amplified from RNA of *E. grandis* stem by RT‐PCR. The PCR products of *EgLBD22*,* EgLBD29* and *EgLBD37* were digested with *Xba*I and *Xma*I and directionally ligated into vector pBI121. All the primers used to construct the overexpression vector are shown in Table S1. Leaf discs from 84k were infected with an overnight culture of *Agrobacterium tumefaciens* harbouring the *35S::EgLBD22*,* 35S::EgLBD29* or *35S::EgLBD37* construct at an OD_600_ of 0.5. Infected leaf discs were then cocultured in darkness with *Agrobacterium* in the shoot induction medium (Murashige‐Skoog basal medium containing 0.5 mg 6‐benzylaminopurine and 0.05 mg naphthalene acetic acid per litre) for 3 days at 23 ± 2 °C. The leaf discs were then transferred to the shoot induction medium containing 200 mg/L Timentin and 40 mg/L Kanamycin under a 16/8 h light/dark regime. After one month, individual regenerated shoots were cut off and transferred to root induction medium (half‐strength Murashige‐Skoog medium supplemented with 0.05 mg/L indole‐3‐butyric acid and 0.02 mg/L naphthalene acetic acid) containing 200 mg/L Timentin and 40 mg/L Kanamycin.

### Phenotype and microscopy analysis

Phenotype analysis for plant height, stem diameter, leaf size and internode length was carried out with ten‐week‐old plants. Five independent lines were used for phenotype investigation for each construct. Significant differences relative to control wild‐type plants (WT‐84k) were determined by Student's *t*‐test. At the same time, stem segments (0.5 cm in length) of the 10th node from WT‐84 and transgenic plants were sampled, immediately fixed in FAA (formaldehyde 3.7%, ethyl alcohol 50.0% and acetic acid 5.0%) and embedded using Shandon Excelior and Histocentre 2 (Thermo Fisher Scientific, Waltham, MA, USA). Five‐μm‐thick sections were stained with safranin and fast green (Gefan biotechnology Co. Ltd., Shanghai). Images were taken using Leica DM 6000B fully automated upright microscope (Leica Microsystems GmbH, Wetzlar, Germany).

### RNA‐seq analysis of transgenic plants

The total RNA was isolated from the shoots of one‐month‐old *EgLBD22‐oe*,* EgLBD29‐oe* or *EgLBD37‐oe* transgenic plants according to the method as described above. RNA integrity was assessed using the Agilent Bioanalyzer 2100 system (Agilent Technologies Santa Clara, CA, USA ). A total amount of 1.5 μg RNA per sample was used for library preparations. Libraries were generated using NEBNext^®^ Ultra™ RNA Library Prep Kit (NEB Ipswich, MA, USA), and index codes were added to attribute sequences to each sample. Library quality was assessed on the Agilent Bioanalyzer 2100 system. The clustering of the index‐coded samples was performed on a cBot Cluster Generation System using TruSeq PE Cluster Kit v3‐cBot‐HS (Illumina San Diego, CA, USA). After cluster generation, the library preparations were sequenced on an Illumina Hiseq platform and paired‐end reads were generated.

Transcriptome assembly was accomplished based on the left.fq and right.fq using Trinity (Grabherr *et al*., 2011) with min_kmer_cov set to 2, and all other parameters set to default values. Gene function was annotated based on Nr (NCBI nonredundant protein sequences), Nt (NCBI nonredundant nucleotide sequences), Pfam (Protein family), KOG/COG (Clusters of Orthologous Groups of proteins), Swiss‐Prot (a manually annotated and reviewed protein sequence database), KO (KEGG Ortholog database) and GO (Gene Ontology) databases. Clean reads were obtained by removing low‐quality reads and reads containing adapter, ploy‐N from raw data and were mapped back to the assembled transcriptome. Readcounts for each gene were obtained from the mapping results and then were adjusted by edgeR program package through one scaling normalized factor (Robinson *et al*., 2010). We estimated the expression levels of each unique sequence in the two samples using RSEM (Li and Dewey, 2011). We then executed statistical analysis using the mapped read numbers for each unique sequences calculated by RSEM. Differential expression analysis was performed using the DEGseq R package (Wang *et al*., 2010). *P* value was adjusted using *q* value (Storey and Tibshirani, 2003). *q* value <0.005 and |log_2_(fold change)| > 1 was set as the threshold. Gene ontology (GO) enrichment analysis of the differentially expressed genes (DEGs) was implemented by the GOseq R package based on Wallenius noncentral hypergeometric distribution (Young *et al*., 2010). Validation of the gene expression was carried out using qRT‐PCR. The specific primers for qRT‐PCR analysis are shown in Table S1.

## Supporting information


**Figure S1** Conserved domains of EgLBD protein family.
**Figure S2** The chromosomal localization of the *LBD* gene family in *Eucalyptus grandis*.
**Figure S3** Subcellular localization of EgLBD22, EgLBD29 and EgLBD37 proteins.
**Figure S4** Gel electrophoresis analysis for the presence of the transgene in *EgLBD22‐oe*,* EgLBD29‐oe* and *EgLBD37‐oe* plants.
**Figure S5** Validation for the expression of the transgene in *EgLBD22‐oe*,* EgLBD29‐oe* and *EgLBD37‐oe* plants by qRT‐PCR.
**Table S1** All the primers used in this study.
**Table S2** The coding sequences of *LBD* genes in *Eucalyptus grandis*.
**Table S3** The information of *LBD* gene family in *Eucalyptus grandis*.
**Table S4** Conserved motifs predicted by MEME program in EgLBD proteins.
**Table S5** Protein‐protein interaction prediction for possible functional protein association networks of EgLBD22.
**Table S6** Protein‐protein interaction prediction for possible functional protein association networks of EgLBD29.
**Table S7** Protein‐protein interaction prediction for possible functional protein association networks of EgLBD37.

**Table S8** The differentially expressed genes between *EgLBD22‐oe* and WT‐84k plants.

**Table S9** The differentially expressed genes between *EgLBD29‐oe* and WT‐84k plants.

**Table S10** The differentially expressed genes between *EgLBD37‐oe* and WT‐84k plants.

**Table S11** The information of eight key differentially expressed genes in *EgLBD22‐oe*,* EgLBD29‐oe* and *EgLBD37‐oe* plants.
