# Supplementary material for: Genomewide analysis of the lateral organ boundaries domain gene family in Eucalyptus grandis reveals members that differentially impact secondary growth
Source: Plant Biotechnol J. 2017 Jul 25;16(1):124–36. doi: 10.1111/pbi.12754 (PMC5785364; doi:10.1111/pbi.12754)
Supplement: Supplementary file 1 — Figure S1 Conserved domains of EgLBD protein family. Figure S2 The chromosomal localization of the LBD gene family in Eucalyptus grandis. Figure S3 Subcellular localization of EgLBD22, EgLBD29 and EgLBD37 proteins. Figure S4 Gel electrophoresis analysis for the presence of the transgene in EgLBD22‐oe, EgLBD29‐oe and EgLBD37‐oe plants. Figure S5 Validation for the expression of the transgene in EgLBD22‐oe, EgLBD29‐oe and EgLBD37‐oe plants by qRT‐PCR. Table S1 All the primers used in this study. Table S2 The coding sequences of LBD genes in Eucalyptus grandis. Table S3 The information of LBD gene family in Eucalyptus grandis. Table S4 Conserved motifs predicted by MEME program in EgLBD proteins. Table S5 Protein‐protein interaction prediction for possible functional protein association networks of EgLBD22. Table S6 Protein‐protein interaction prediction for possible functional protein association networks of EgLBD29. Table S7 Protein‐protein interaction prediction for possible functional protein association networks of EgLBD37. [file PBI-16-124-s003.pdf]

Supporting information

Genome-wide analysis of the lateral organ boundaries domain (LBD) gene family in *Eucalyptus grandis* reveals members that differentially impact secondary growth

Qiang Lu, **Fenjuan Shao**, **Colleen Macmillan**, **Iain W. Wilson**, **Karen van der Merwe**, **Steven G. Hussey**, **Alexander A. Myburg**, **Xiaomei Dong**, **Deyou Qiu**

Figure S1-S5

Table S1-S11

Figure S1 Conserved domains of EgLBD protein family. (a) Sequence alignment of EgLBD proteins by DNAMAN. (b) The $CX_2CX_6CX_3C$ zinc finger-like motif logos and the $LX_6LX_3LX_6L$ leucine zipper-like motif logos.

Figure S2 The chromosomal localization of the *LBD* gene family in *Eucalyptus grandis*. The scale bar represents 8.0 Mb chromosomal distance, the chromosome number (Chrom01-Chrom11) is indicated at the top of each chromosome, sister paralogous pairs are connected by dotted lines.

Figure S3 Subcellular localization of EgLBD22, EgLBD29 and EgLBD37 proteins. The photographs were taken under dark-field illumination for green fluorescence localization (green fluorescent), cells of tobacco seedlings expressing *EgLBD22/29/37*-GFP were stained with DAPI (5 ug/ml) to show the nuclear localization (DAPI stains nucleus, blue fluorescent), under bright-field illumination to examine cell morphology (bright-field), and under merged-field illumination (merged). From top to bottom line is *EgLBD22, EgLBD29, EgLBD37* and *pCAMBIA1302-GFP* (the positive control) respectively. The bar was 92 μm.

Figure S4 Gel electrophoresis analysis for the presence of the transgene in *EgLBD22-oe*, *EgLBD29-oe* and *EgLBD37-oe* plants. (a) Gel electrophoresis result for *EgLBD22-oe* line 1, 2, 3, 4, 5, 6, 7, 8, 9, 10, 11, 12, 13, 14, 15, 16, 17, 18, 21 and CK (WT-84k). (b) Gel electrophoresis result for *EgLBD29-oe* line 1, 2, 4, 6, 7, 9, 10, 11, 25, 28, 34, 37, 40, 42, 45, 47 and CK (WT-84k). (c) Gel electrophoresis result for *EgLBD37-oe* line 1, 2, 3, 4, 5, 6, 7, 8, 10, 11, 12, 13, 14, 15, 16, 17 and CK (WT-84k).

Figure S5 Validation for the expression of the transgene in *EgLBD22-oe*, *EgLBD29-oe* and *EgLBD37-oe* plants by qRT-PCR. (a) qRT-PCR result for *EgLBD22-oe* line 1, 3, 4, 6, 7, 8, 11, 12, 15, 17, 18, 21 and CK (WT-84k). (b) qRT-PCR result for *EgLBD29-oe* line 1, 2, 4, 5, 6, 7, 9, 10, 11, 13, 25, 45 and CK (WT-84k). (c) qRT-PCR result for *EgLBD37-oe* line 1, 2, 3, 4, 5, 6, 7, 8, 10, 11, 12, 13 and CK (WT-84k).

Table S1 All the primers used in this study.

Table S2 The coding sequences of *LBD* genes in *Eucalyptus grandis*.

Table S3 The information of *LBD* gene family in *Eucalyptus grandis*.

Table S4 Conserved motifs predicted by MEME program in EgLBD proteins.

Table S5 Protein-protein interaction prediction for possible functional protein association networks of EgLBD22.

Table S6 Protein-protein interaction prediction for possible functional protein association networks of EgLBD29.

Table S7 Protein-protein interaction prediction for possible functional protein association networks of EgLBD37.

Table S8 The differentially expressed genes between *EgLBD22-oe* and WT-84k plants.

Table S9 The differentially expressed genes between *EgLBD29-oe* and WT-84k plants.

Table S10 The differentially expressed genes between *EgLBD37-oe* and WT-84k plants.

Table S11 The information of eight key differentially expressed genes in *EgLBD22-oe*, *EgLBD29-oe* and *EgLBD37-oe* plants.

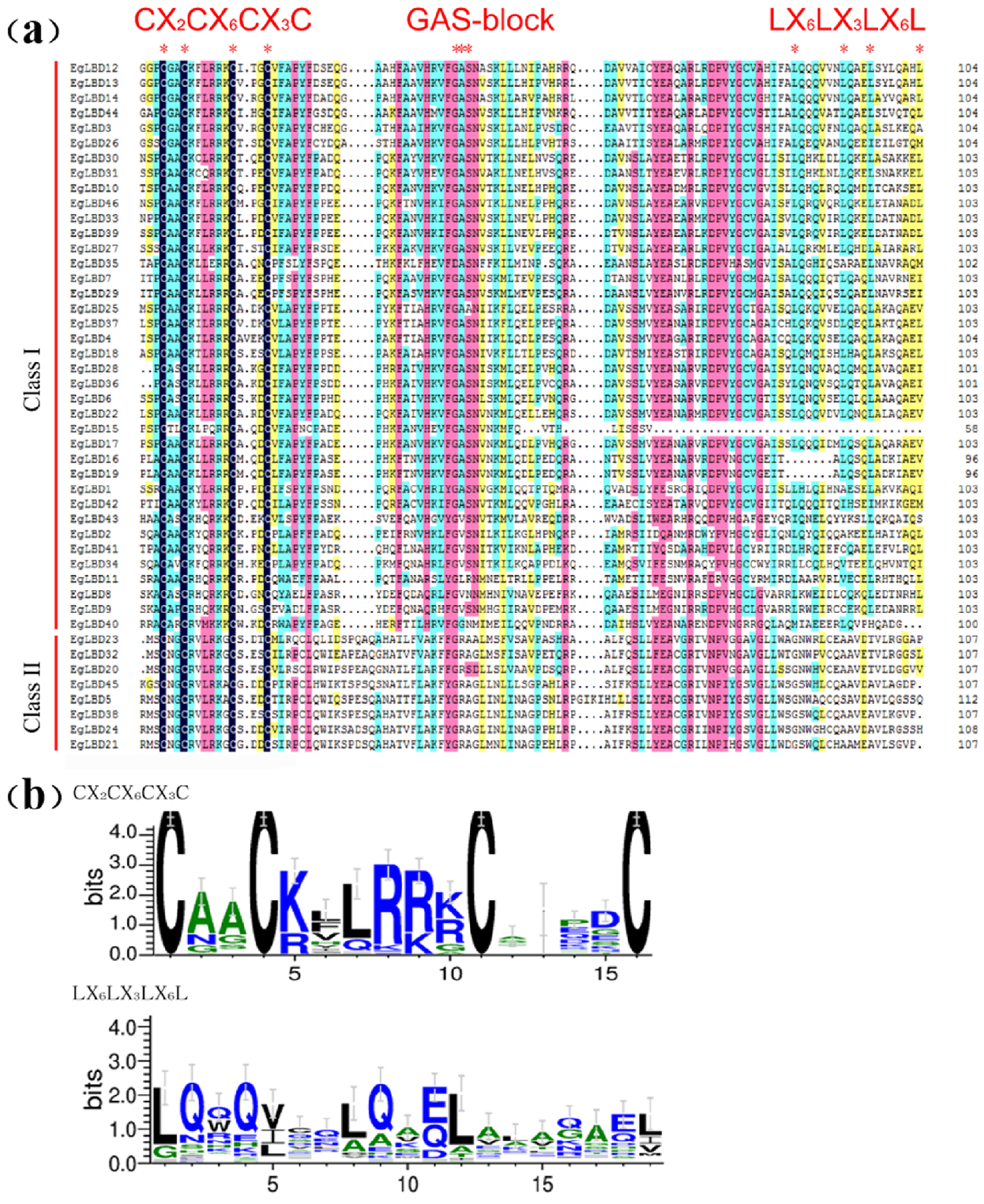

Figure S1. Conserved domains of EgLBD protein family. (a) Sequence alignment of EgLBD proteins by DNAMAN. (b) The $CX_2CX_6CX_3C$ zinc finger-like motif logos and the $LX_6LX_3LX_6L$ leucine zipper-like motifs logos.

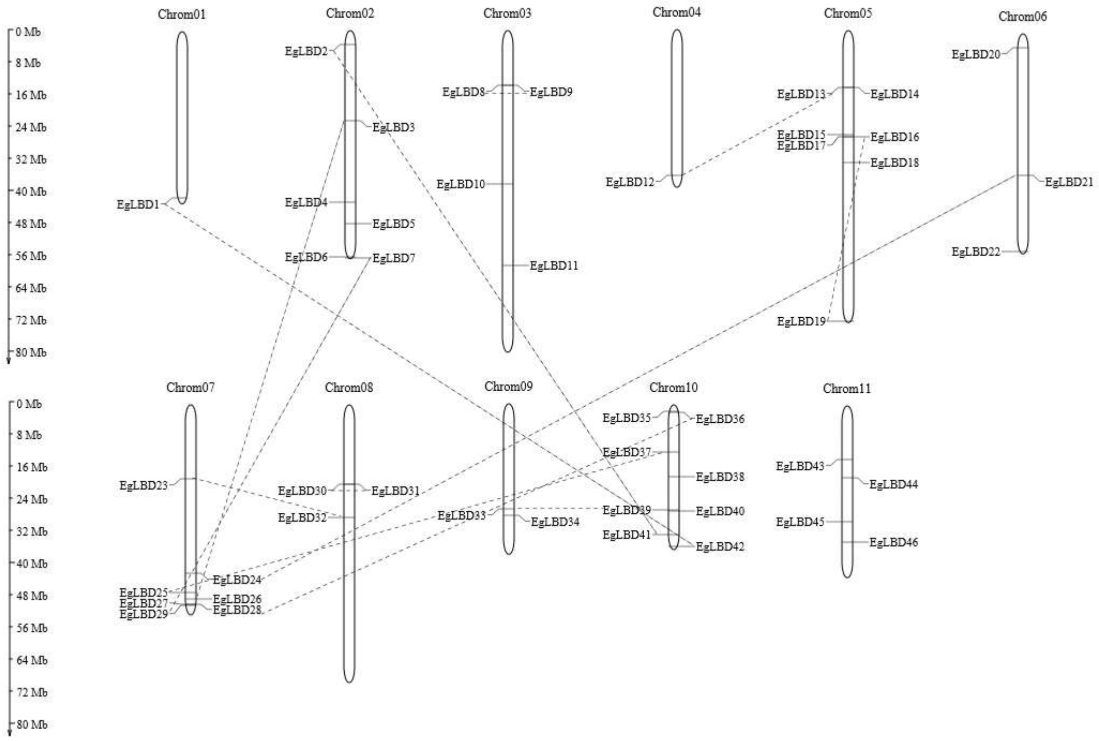

Figure S2 The chromosomal localization of the *LBD* gene family in *Eucalyptus grandis*. The scale bar represents 8.0 Mb chromosomal distance, the chromosome number (Chrom01-Chrom11) is indicated at the top of each chromosome, sister paralogous pairs are connected by dotted lines.

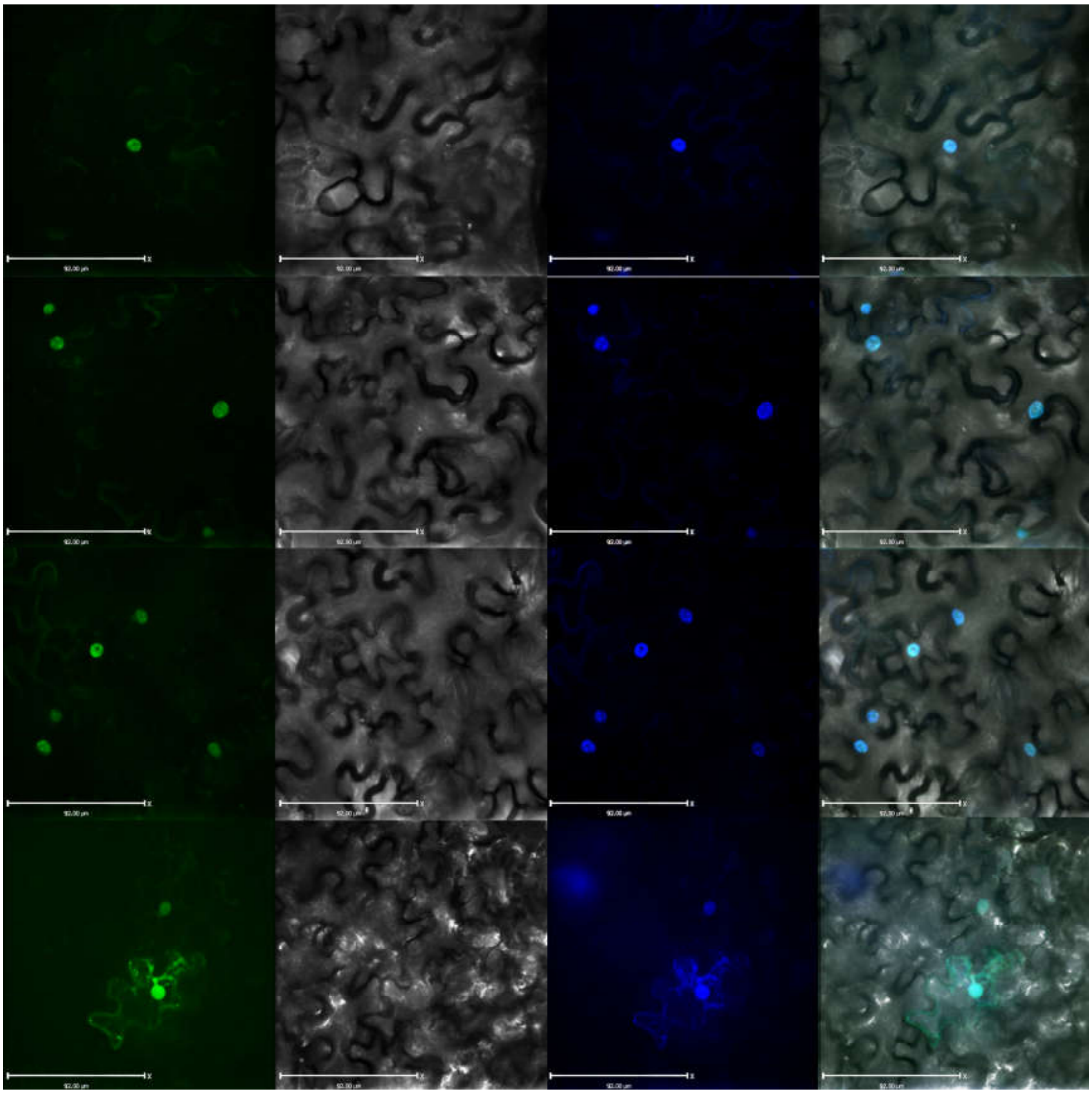

Figure S3. Subcellular localization of EgLBD22, EgLBD29 and EgLBD37 proteins. The photographs were taken under dark-field illumination for green fluorescence localization (green fluorescent), cells of tobacco seedlings expressing *EgLBD22/29/37*-GFP were stained with DAPI (5 ug/ml) to show the nuclear localization (DAPI stains nucleus, blue fluorescent), under bright-field illumination to examine cell morphology (bright-field), and under merged-field illumination (merged). From top to bottom line is the signal of proteins encoded by *EgLBD22*, *EgLBD29*, *EgLBD37* and *pCAMBIA1302-GFP* (the positive control) respectively. The bar was 92 μm.

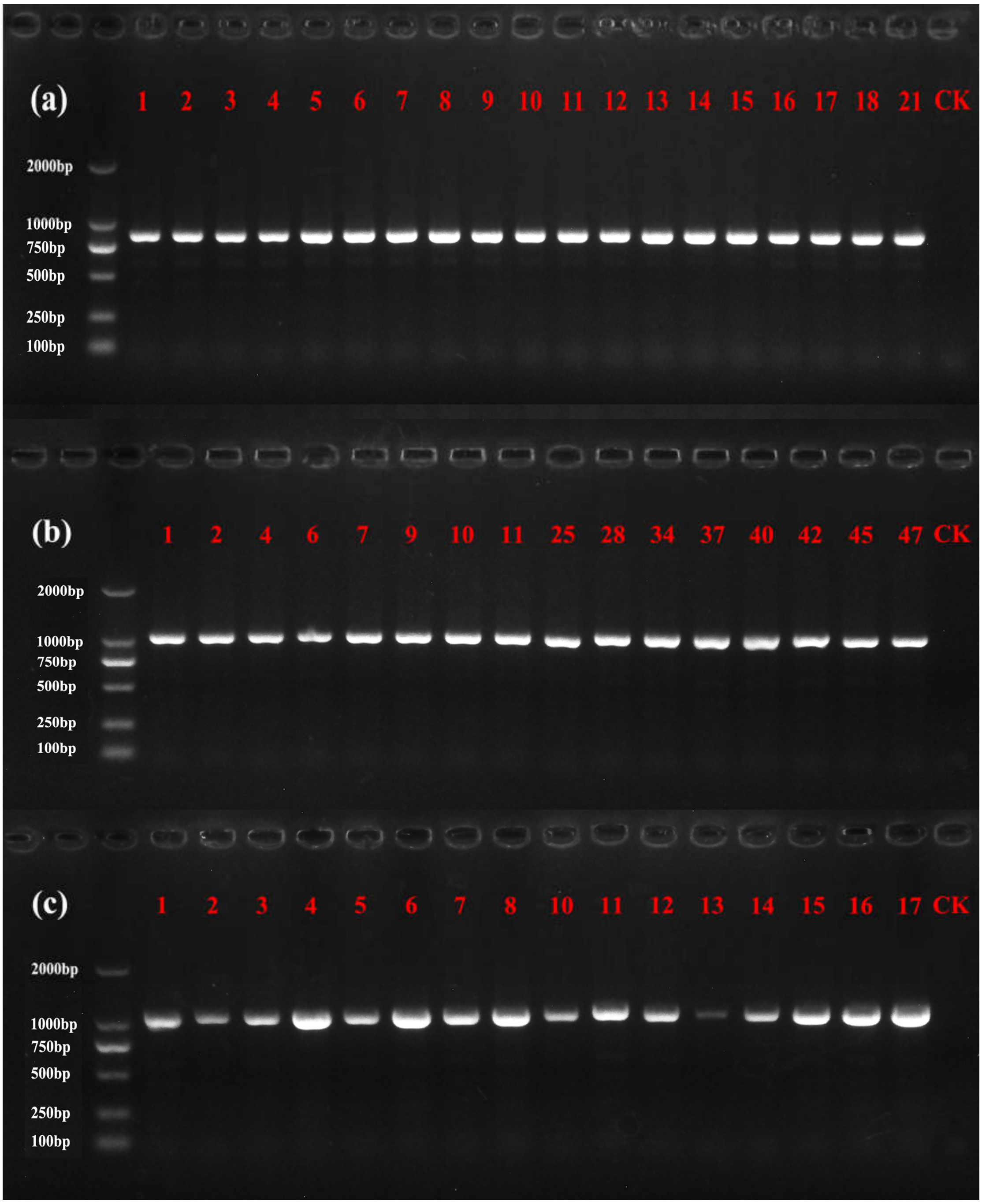

Figure S4 Gel electrophoresis analysis for the presence of the transgene in *EgLBD22-oe*, *EgLBD29-oe* and *EgLBD37-oe* plants. (a) Gel electrophoresis result for *EgLBD22-oe* line 1, 2, 3, 4, 5, 6, 7, 8, 9, 10, 11, 12, 13, 14, 15, 16, 17, 18, 21 and CK (WT-84k). (b) Gel electrophoresis result for *EgLBD29-oe* line 1, 2, 4, 6, 7, 9, 10, 11, 25, 28, 34, 37, 40, 42, 45, 47 and CK (WT-84k). (c) Gel electrophoresis result for *EgLBD37-oe* line 1, 2, 3, 4, 5, 6, 7, 8, 10, 11, 12, 13, 14, 15, 16, 17 and CK (WT-84k).

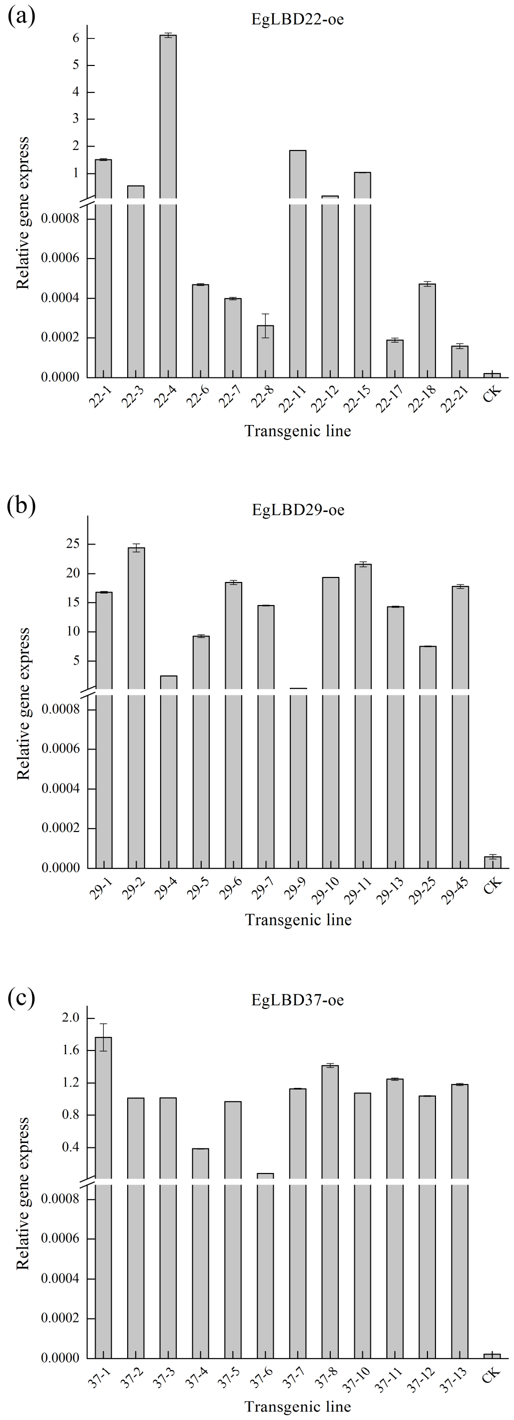

Figure S5 Validation for the expression of the transgene in *EgLBD22-oe*, *EgLBD29-oe* and *EgLBD37-oe* plants by qRT-PCR. (a) qRT-PCR result for *EgLBD22-oe* line 1, 3, 4, 6, 7, 8, 11, 12, 15, 17, 18, 21 and CK (WT-84k). (b) qRT-PCR result for *EgLBD29-oe* line 1, 2, 4, 5, 6, 7, 9, 10, 11, 13, 25, 45 and CK (WT-84k). (c) qRT-PCR result for *EgLBD37-oe* line 1, 2, 3, 4, 5, 6, 7, 8, 10, 11, 12, 13 and CK (WT-84k).

Table S1 All the primers used in this study.

	Gene Name	Primer Sequence (5'…3')
**Primers used in qRT-PCR analysis for tissue-specific expression of *EgLBD* genes and their response to GA and IAA treatment**		
	EgLBD1-F	TCTGATGCTAATCGGGAACC
	EgLBD1-R	GAAGAGCCTATCTGATGCTGC
	EgLBD2-F	CTAGCTCCTTTCTTCCCGGC
	EgLBD2-R	GGTACCAATCGCGCATGTTC
	EgLBD3-F	TGAACGCAATGCCTTACTATGA
	EgLBD3-R	CCGTCCCGGAAACTCCA
	EgLBD4-F	GAGCCCGCCAAGTTCACTAT
	EgLBD4-R	TCGCTCACTTGCTTCTGGAG
	EgLBD5-F	TCTACGAGGAGACGAGCCA
	EgLBD5-R	CCCAGGTTTGCCAGTGAA
	EgLBD6-F	ACCCCACGACCCTCATAAGT
	EgLBD6-R	TCACCGCATCTCCTCTCTGA
	EgLBD7-F	GAAGAGTGCCCTTTCTCCCC
	EgLBD7-R	TGTTGCAAAGCCGAGATTGC
	EgLBD8-F	CGACCACTACTCTGCTCGAC
	EgLBD8-R	TCATTCCATGGCCCTGTCAC
	EgLBD9-F	GAGGGTAACATCAGGAGACG
	EgLBD9-R	CAAAGACAGGCGACGATT
	EgLBD10-F	CACCAGTTCTTTCCCCGTGA
	EgLBD10-R	GAGAAGGCTTGCGTCGTAGT
	EgLBD11-F	CGGAGCGTCGACTTCATCAT
	EgLBD11-R	GCATGCCAAGTTGAGGATCG
	EgLBD12-F	TGCTCATTACCAACCACCAT
	EgLBD12-R	GTGAAGTATCAGGCATTCGG
	EgLBD13-F	TATTCGCGCCCTACTTCGAC
	EgLBD13-R	CGCCTCGTAGCATATGGTGA
	EgLBD14-F	CAGCACTTCCCTCTACAGCC
	EgLBD14-R	ATGTTTGGCAGAGTCGGGTT
	EgLBD15-F	GGCTGGAAGCAGGGGATG
	EgLBD15-R	CGTTGGCGAACTTGTGGG
	EgLBD16-F	GAGCCCCACAAGTTCACCAA
	EgLBD16-R	CCAGGCTACTCACGGTGTTT
	EgLBD17-F	ATGAAGGAAAGCGGTGGGAG
	EgLBD17-R	CCGGGAAATATGGGGCAAAC
	EgLBD18-F	TGACACTTCCCGAATCGCAA
	EgLBD18-R	CGTGAGATTAGCCCGTTGGA
	EgLBD19-F	GAGCCCCACAAGTTCACCAA
	EgLBD19-R	CCAGGCTACTCACGGTGTTT
	EgLBD20-F	CAAGAGGAGCCACGTGACAA
	EgLBD20-R	GGTTCAGGAGCTTCGTCTCT
	EgLBD21-F	CGAGCTGACGCTAGGGTTCAA

	EgLBD21-R	CATGCTGCACGCTTCGTCTCT
	EgLBD22-F	GCCAGCAATGTCAACAAGAT
	EgLBD22-R	ATACAGGGTCTCGCATCCTT
	EgLBD23-F	GACCACTTTAGGCCTCGGAG
	EgLBD23-R	AGACATCAGGGACAGGTCGA
	EgLBD24-F	GCCCTGCCTACAATGGATCA
	EgLBD24-R	TGATGAGGTTGATGAGGCCG
	EgLBD25-F	GCAGTGCCAGAACACCAA
	EgLBD25-R	CCCAAGAAGGGATGAGGT
	EgLBD26-F	TTGGCTCTTCATGTGGTGCA
	EgLBD26-R	GGCCTGGTCGTAGCAGAAAT
	EgLBD27-F	ATGAGGGCGATGCGAATGAA
	EgLBD27-R	TCGACGTGCACTTCCTCTTC
	EgLBD28-F	AGCCTTGTGTACGAAGCCAA
	EgLBD28-R	CTTGTCGTCTGAGCTGTCCA
	EgLBD29-F	TGAGAGCCAAAGAGCTGACG
	EgLBD29-R	GGGACTGATGATGCCTGCTT
	EgLBD30-F	GACAGCAGCAACAGCAACAA
	EgLBD30-R	CGGCGGTGGAGAGATTACAT
	EgLBD31-F	TCGCACAAATGGTTCTGACT
	EgLBD31-R	CTTGCTCGTGGTATGGGTT
	EgLBD32-F	AGGGTTCTGAGGAAGGGCT
	EgLBD32-R	AACTTGGCGACGAAGACG
	EgLBD33-F	TCAACTCTCTCGCCTATGAGG
	EgLBD33-R	GCTCCTTCTGTAGCCTGATGA
	EgLBD34-F	GTCCCGACCCGACAATCTTT
	EgLBD34-R	TGAACACGTCCGCACCATAA
	EgLBD35-F	TGCAAGGCCACATTCAGAGT
	EgLBD35-R	AGCAGCGATGTTAGGCAGAA
	EgLBD36-F	GGACTGCATCTTCGCTCCTT
	EgLBD36-R	TCTGACAAACCGGAAGCTCC
	EgLBD37-F	CCCACCGAGCCTTACAAGTT
	EgLBD37-R	CTCGTACACCATGCTGCTCA
	EgLBD38-F	GATGAGCTGTAACGGGTGCA
	EgLBD38-R	AGAACTTGGCGAGGAAGACG
	EgLBD39-F	GGAGAAAGTGCCTGCCTGAT
	EgLBD39-R	TCACGTTGCTAGCCCCAAAT
	EgLBD40-F	GAACGCTTCACCATCCTCCA
	EgLBD40-R	TCAGCAATCATCTGGGCGAG
	EgLBD41-F	CCCGTCGGACTTTCAGATCA
	EgLBD41-R	TCCTGTCGTCTGCAATGTCC
	EgLBD42-F	CTGTCTATGGATGCGTTGGA
	EgLBD42-R	ATGGAAGGTGTTGTGGGTGT

	EgLBD43-F	TGCAAGGGGAGATCAGCAAG
	EgLBD43-R	CGGTATTCATGCTCGTCCCA
	EgLBD44-F	CGTCAACAAGAGGCAGGATG
	EgLBD44-R	GCAATGCAAGGATGGTCGAT
	EgLBD45-F	GAGGTTGGAGCCGAGAAGAG
	EgLBD45-R	GAACGTCCAGACCAGAGCTC
	EgLBD46-F	CCAATGCCGATCTCATCCGA
	EgLBD46-R	CCTCCCAACACCACCAAACT
	PP2A3--F	CAGCGGCAAACAACTTGAAGCG
	PP2A3--R	ATTATGTGCTGCATTGCCCAGTC
**Primers used in subcellular localization analysis**	EgLBD22-GFP-F	AGTTCATTTCATTTGGAGAGAACACGGGGGACTCTTGACCATGAAAGAGAGTGGA
	EgLBD22-GFP-R	TTGGGACAACTCCAGTGAAAAGTTCTTCTCCTTTACTAGTTGACCACAAAGAGTC
	EgLBD29-GFP-F	AGTTCATTTCATTTGGAGAGAACACGGGGGACTCTTGACCATGTCCAGAGAAAGT
	EgLBD29-GFP-R	TTGGGACAACTCCAGTGAAAAGTTCTTCTCCTTTACTAGTACCAAAATAAGAGAC
	EgLBD37-GFP-F	CATGCCATGGAAATGGAATACAAGGCGAGAAC
	EgLBD37-GFP-R	CTAGACTAGTCGTCCAGTAAATCGATTCCCAG
**Primers used in constructing the overexpression vector**	121-EgLBD22-F	GCTCTAGAATGAAAGAGAGTGGAAGAAAAC
	121-EgLBD22-R	TCCCCCCGGGTCATGACCACAAAGAGTCCTCG
	121-EgLBD29-F	GCTCTAGAATGTCCAGAGAAAGTAGGGAGA
	121-EgLBD29-R	TCCCCCCGGGTTAACCAAAATAAGAGACATTC
	121-EgLBD37-F	GCTCTAGAATGGAAATGGAATACAAGGCGA
	121-EgLBD37-R	TCCCCCCGGGTCACGTCCAGTAAATCGATTCC
**Primers used in analysis for the presence of the transgene**	EgLBD22-F	GCCATCATTGCGATAAAGGAAA
	EgLBD22-R	ATCCAGACTGAATGCCCACAGG
	EgLBD29-F	GCCATCATTGCGATAAAGGAAA
	EgLBD29-R	ATCCAGACTGAATGCCCACAGG
	EgLBD37-F	GCCATCATTGCGATAAAGGAAA
	EgLBD37-R	ATCCAGACTGAATGCCCACAGG
**Primers used in validation of the RNA-seq results for the expression of selected genes in the transgenic plants**	auxin efflux carrier component 6-F	TCCCCATCACGGTGTTAGTTAA
	auxin efflux carrier component 6-R	ACATATGGCTCAAAATCAGGGC
	expansin gene (c60809_g2)-F	TGATGGTAGGACTGTGACTAGC
	expansin gene (c60809_g2)-R	CTTTCCTACCCTACCCCTTCTC

	ERF023-F	TTGGAATCAGTACCGAATGTGC
	ERF023-R	TGGCAGCGTATTGTCTTAAAGG
	ethylene-responsive element-binding (EREB) family protein -F	AATGTTCCAAGCCATACACGAG
	ethylene-responsive element-binding (EREB) family protein -R	ACCAAAGTCTCCAAGCACTCTA
	expansin (c60754_g1)-F	ATGAGATGTGATGATGACCCCA
	expansin (c60754_g1)-R	GCACCCTTCTAAAAGCTACAGG
	gibberellin-regulated protein 5-F	ACCAAGATGTAATTACCGCTGC
	gibberellin-regulated protein 5-R	CCTTGTTGCCATAAGTACCAGG
	expansin-B3-F	GGTCATTAGTGGATGTGAAGCC
	expansin-B3-R	AAAGTGAGTGTTGCCATTGGAA
	MYB Family APL-like-F	TCACAGAACTTGAATGACGACG
	MYB Family APL-like-R	CCTGTGTCTGTCCCTCTCTATC
	PtActin-F	AACTGTAATGGTCCTCCCTCCG
	PtActin-R	GCATCATCACAATCACTCTCCGA
**Primers used in analysis for the expression of selected genes in the transgenic plants**	EgLBD22-F	GCCAGCAATGTCAACAAGAT
	EgLBD22-R	ATACAGGGTCTCGCATCCTT
	EgLBD29-F	TGAGAGCCAAAGAGCTGACG
	EgLBD29-R	GGGACTGATGATGCCTGCTT
	EgLBD37-F	CCCACCGAGCCTTACAAGTT
	EgLBD37-R	CTCGTACACCATGCTGCTCA
	PtActin-F	AACTGTAATGGTCCTCCCTCCG
	PtActin-R	GCATCATCACAATCACTCTCCGA

Table S2 The coding sequences of *LBD* genes in *Eucalyptus grandis*.

GenBank accession number	Euc ID	Gene name	Coding Sequence
XP_010025110	Eucgr.A02814.1	*EgLBD1*	ATGTCAACCATTTCATCTCGATGTGCAG CTTGCAAATATTTGAGAAGAAGATGCCC TCCAGATTGCATTTTCTCTCCTTATTTCC CATCCAATGACCCCCAAAGATTTGCTTG TGTTCACAGGATCTATGGTGCGAGCAAT GTTGGCAAAATGATCCAGCAAACTCCG ACCCAAATGAGAGCTCAAGTTGCGGATT CGCTGTACTTCGAGTCACGATGCCGGAT TCAAGACCCGGTGTACGGATGTGTCGG GATCATCTCCCTGCTGCATTTACAAATAC ATAATGCCGAAAGCGAATTGGCTAAAGT AAAAGCACAAATCGCGTTTCTTAATTCT GATGCTAATCGGGAACCGAGTTTCCGAC CACAGCTCGAGGGTGCTTCCAATGTTAA CAATTTATGGAGTGCACAAAGCAGTGTT GAGCAGCATCAGATAGGCTCTTCTACAA CTCATGGTCCTTTAGCGTTCATGTAG
XP_010028622	Eucgr.B00397.1	*EgLBD2*	ATGACCCTGAAGGGTGGCGCCAGCCAG GCATGCGCGGCGTGCAAGTACCAGAGG AGGAAGTGCAAACCAGACTGCCCTCTA GCTCCTTTCTTCCCGGCCGACCAGCCGG AGATCTTCCGGAACGTGCACAAGTTGTT TGGCGTGAGCAACATCCTAAAGATATTG AAAGGCCTGCATCCTAACCAGAAGCCC ATCGCCATGAGATCCATCATCGACCAAG CGAACATGCGCGATTGGTACCCGGTGCA CGGCTGCTACGGCCTCATCCAGAACCTG CAGTACCAAATACAGCAGGCCAAGGAA GAGCTCCATGCCATTTACGCGCAGCTAG AGATGTACAGGCAACAGAACCAGGTCT CTCCGGTCCCTGAAGACGTGCCTTCTCA GCTGGAATTGGGCATGGCCCCACCAAA CGGCGCGCTTCCCCTGTTCCACCAAACG CAGCAGCAGTGCAGCGCTGCGGCCGCC TTGCCCGTTCCGCAAGATCACTGCGGTA ACAACGGAGGCGTAGCTGGTTACGACA GCTCCAGTGATGTGGTGCCCAAGGATAA

			TGTCGAGAATGCCTTGTGGATGCAAAAT<br>CAATACGCTAATTACAGCAACAGCAATG<br>CAATTCAGATGGCTGCATCGCAGCCGAT<br>GGACGTGCAGCAAGAAATAGTCCAAGA<br>CTACGATGAACTCCAACCTTTCTTCGAC<br>ACCATCGACGATACACAGTCTTATAAAG<br>AGGCATGCGACTCGAGCTCGGAATCGTC<br>ATTGAAGGATACAAGGCAATCCATCGAG<br>CATGCTGGCAACAACGATTTGAAGAGC<br>GCTGCCGCATGCTTCAGCCTCACCAGTG<br>TCAACTGA
XP_010039<br>047	Eucgr.B01529.1	*EgLBD3*	ATGACTGGTTCTGGTTCTCCTTGTGGCG<br>CTTGCAAGTTCTTGAGGAGGAAATGCGT<br>GAGAGGCTGCGTCTTTGCTCCATATTTTT<br>GCCACGAGCAAGGTGCGACGCATTTCG<br>CGGCCATTCACAAGGTCTTTGGCGCAAG<br>CAACGTGTCAAAGCTCCTGGCAAACCT<br>CCCCGTGAGTGATCGGTGCGAGGCCGC<br>CGTGACCATCTCGTATGAGGCTCAGGCG<br>CGGCTGCAGGACCCCATTTATGGATGCG<br>TCTCGCACATCTTTGCGCTCCAACAACA<br>GGTATTCAATTTGCAAGCGCAATTGGCT<br>TCTCTGAAGGAGCAAGCTACTCAAAGC<br>CTTTGTGCTCCCTCTTCCACGAGAGGGA<br>GCCTTAACGAGAAATATTCTGGAAATAA<br>GGTCATCTCGAACCAAGCCCTTGACGTG<br>CAAAGTTGGTTGCACATGGCAAATCCAA<br>ATAATTTGCCTCATCAGTTCAGTTTAGAT<br>AGCATGAGCAATGTGAACGCAATGCCTT<br>ACTATGACAATGGGTCGATGGATTCAAG<br>CTCTGCAGGGAACAATTATGACAATTCA<br>CTTGTCCTAGAAGAGAACATCTCCTTTG<br>GGAGCTTAGAAGAGGCTTCTGGTGGTT<br>CGATCTCTTCAATGGATATGCAAACAAG<br>CAACAGGCAGTGGAGTTTCCGGGACGG<br>TGATGACCTCCACTCCTTCACGTATGGG<br>TACATTCACCATTAG
XP_010043<br>701	Eucgr.B02465.1	*EgLBD4*	ATGGCTGTGCAAGATATGGAGGTTTCGG<br>ACGCGAGTGTCACGAACGGAAACGCCC<br>ACACGAGCTCCACAACCACCTCTTCTCC<br>GCAGTCTGTCTCGCCTACTTCGTCGTCT<br>TCTCCTCCTCCCCCTCTTCCTCCGCCGA<br>CGCCGGTCATCATAAGCCCTTGCGCGGC<br>CTGCAAGATTCTGAGGCGAAGGTGTGC

			CGTCGAGAAGTGCGTGCTGGCGCCCTAT<br>TTTCCTCCCACCGAGCCCGCCAAGTTCA<br>CTATTGCCCATCGCGTCTTCGGCGCAAG<br>CAACATCATCAAGCTCTTGCAGGACCTT<br>CCAGAATCACAGCGAGCCGATGCGGTG<br>AGTAGCATGGTGTATGAGGCTGGGGCGA<br>GGATCAGGGACCCTGTCTATGGCTGCGC<br>AGGCGCGATCTGCCAACTCCAGAAGCA<br>AGTGAGCGAGCTCCAGGCTCAGCTGGC<br>CAAGGCCCAAGCCGAGATCCTCACCAT<br>GCAACACCAGCAAACCAACCTGGTGGC<br>CTTGCTGTGCATGGAAATGGCTCAGTCG<br>GCTTCTCAGCCGCCAGCGCAACAATTCG<br>TCGACAATCTAATCGACACCAGTCCAAC<br>CATCTACCAGAACAACAGTAACAACAA<br>CATAAGCCCGTTCCTGGACGAGACCAA<br>CGTCATGGGGTCCATGTGGGAGCCGCTT<br>TGGACATGA
KCW86326	Eucgr.B03016.1	*EgLBD5*	ATGAGAATGAGCTGCAACGGGTGTAGA<br>GTCCTCCGGAAAGCCTGCAGCGAAGAT<br>TGCACCATCAGGCCTTGCCTCCAGTGGA<br>TCCAATCCCCCGAGTCGCAGGCCAACG<br>CCACCACGTTCCTCGCCAAGTTCTACGG<br>CCGCGCCGGCCTCATCAACCTCATCAAT<br>GCTGGCCCCTCCAACCTCCGCCCAGGTA<br>TCAAAATCCATCTACTTCTGTCGTTGCTG<br>TACGAGGCCTGCGGGCGGATCGTCAAC<br>CCGATCTACGGCTCGGTCGGGCTGCTCT<br>GGTCGGGCAACTGGGCCCAGTGCCAGT<br>CGGCCGTCGATGCGGTGCTCCAGGGCTC<br>CTCCCAGTTCTTGCGATCGCCTTCGCTC<br>GACTCCACGGGGCCCCGCCTCAAGCCC<br>TGCGACATCCGCCACGTCCGGAAGACG<br>TCCGGCTCCGGCGGCCTCCGCAAGATCA<br>GGACCGGGACTCAGTTCAAGCGGTCGT<br>CATCGAATCGGGACAAGCTGGGCACGG<br>AGCCCGAGGAGCTGTTCGGCCGTGAAG<br>GGAGCAAGTGGATCTACCCGGGCCCCAT<br>CTACGAGGAGACGAGCCACGACTCACG<br>GGCGGCGGCGAACCGGGAGAGCGCGG<br>AGGCCGAGAGCATCTTCTCGGTGGAGA<br>CCGTGGAGGCTTCACTGGCAAACCTGG<br>GGAAGCGGAACCGGATGCTGAAATTCG<br>ACTGGCCGGCGGAGGAGAGTCCGGAGA

			GTCCACTCCAGTTAGAGCTGGGTCTGGG<br>CGTCAAACCGTCGTCACATCCAAGCGA<br>GCCCGACTTCGGTCGGCATTGA
XP_010045<br>372	Eucgr.B03991.1	*EgLBD6*	ATGGGAGGATCGTCGGGATCTTCGCCAT<br>GTGCTTCCTGCAAGCTGTTGAGACGCCG<br>CTGCTCCAAGGATTGCATCTTCGCTCCAT<br>ATTTCCCACCCCACGACCCTCATAAGTT<br>TGCCATTGTTCATAAGGTCTTTGGTGCC<br>AGTAATCTCAGCAAAATGTTGCAGGAAC<br>TTCCGGTGAATCAGAGAGGAGATGCGG<br>TGAGCAGTTTAGTGTACGAGGCAAATGC<br>AAGGGTGAGAGACCCAGTATATGGGTG<br>CGTAGGCACGATATCTTACTTGCAGAAT<br>CAAGTGTCTGAATTGCAGCTTCAGCTTG<br>CAGCGGCCCAAGCAGAAGTTCTTTCCAT<br>GCAAATGCACCAAAAGCCACATCTTGTA<br>CAACAAGATAGGGAGACGTCTTTTCTCA<br>TGGCTGGGGACAACTTCAACAGCGTGT<br>CACACTATATTGCCGCCAGTGTAATCCA<br>GGATCCTCTTAAGAGAGAGTCCTTTTCG<br>ACTTAA
KCW87571	Eucgr.B04019.1	*EgLBD7*	ATGATGGGGGTGGGAGGTGCTAGGGAA<br>AGATTGGATGAGATAGCAAAGAAGATC<br>AAGAGAGAGAAGGATGCAACTTATGGT<br>GATGAAATGGGAAGAAGGCACATGATG<br>TTGAGCCTAAGCAGAACCGGAACGCTC<br>AACACGATAACGCCGTGCGCGGCGTGC<br>AAGCTTTTGAGGAGGCGATGTGCCGAA<br>GAGTGCCCTTTCTCCCCTTATTTCTCTCC<br>ACACGAGCCTCAGAAATTCGCCGCTGTT<br>CACAGAGTCTTCGGTGCAAGCAACGTC<br>TCCAAGATGCTCACGGAGGTACCGGAG<br>AGCCAAAGAGCTGATACCGCCAACAGT<br>TTGGTGTACGAAGCAAATCTGAGGCTG<br>AGAGACCCAGTGTACGGATGCATGGGT<br>GCAATCTCGGCTTTGCAACAGCAGATCC<br>AGACTCTGCAGGCTCAACTCAACGCTGT<br>AAGAAATGAGATACTGAGATTCAAATAC<br>AGAGAAGCCGTCACTACAAATCACGTA<br>CAATCCAATGTTGTCGTTCCTCATTCTAG<br>AACTGCTCTTAATCCTACTGCTGCTTTGG<br>CTGTTTCCGTTGTTCCTCCTCCAACCCTT<br>TCTTCGTCTTCTTCCACTGCCGCACCTC<br>CCGACCCCTCTCCTCCTCCTCTGCCGCC

			ACCCCCGTCTTTAGTCGTGCCCTCATCTTCTTCCTCCTCCTCTTCCACTTCTTCTTTATTCACTACCCCAACCAGCACCACCACCTATAGCTCCATCTCTAGCGAAACTAATACTGTCCATTACTTCGAAACGATCTGA
XP_010050454	Egrandis_v1_0.045990m.g	*EgLBD8*	ATGCCTGCAGCTTCCAAAGCATGCGCCTCGTGTCGACACCAGCGGAAGAGGTGTGACGGCAACTGCCAGTACGCTGAGCTCTTCCCGGCAAGCCGGTACGACGAGTTCCAGGACGCGCAGCGGCTCTTCGGCGTGAACAACATGCACAACATCGTGAACGCGGTCGAGCCCGAGTTCAGGAAGCAGGCCGCCGAGTCGATACTCATGGAGGGCAACATCAGGAGAAGCGATCCCGTGCACGGGTGTCTCGGCGTTGCTCGGAGGCTCAAGTGGGAGATAGACCTGTGCCAGAAGCAGCTCGAGGACACGAACCGTCACCTATCTTTCCTCCAGCAGAGGGACCGAGAATTGCAGAGGCAACGGCAAGGGTTTAATGATACTCTTGTTGAACTCCCGCCGTTGCTGCCAGCGTTCTTCTCGAATGGGGGCGGCGTCAGGAGTTTCTATGAGCAGATGCAGCGTAACCCGACCACTACTCTGCTCGACTCGAGTTGCATGAATTATCAAGGACTCAATTACCGATCGACGTACGAAGAAGGAGCGACCAGCACTGAGCCCTTCGATCTCTTTCGCGTGACAGGGCCATGGAATGACAACCCGGCCGTGACAATCGCGAGGGCGGACCAGATTACTAAACCTGAAGAGGCGAAGCAGGAGGATTCACGAAGGGTGTGCGATGATGGAACCGAGAGCGTGGAGCTACGTTTGAAAGACCGGGACTTTGAATGTTGTGGCGATCAAGGCAAATAA
XP_010050456	Egrandis_v1_0.037898m.g	*EgLBD9*	ATGCCTGCAACTTCCAAAGCATGCGCACCGTGTAGACACCAAAAGAAGAGGTGCAACGGAAGCTGCGAAGTCGCCGACCTCTTCCCGGCGAGCCGGTACGACGAGTTCCAGAACGCACAGCGGCACTTCGGCGTGAGCAACATGCATGGCATCATACGCGCGGTCGACCCCGAGATGAGGAAACAAGCTGCCGAGTCGATACTCATGGAGGGTAACATCAGGAGACGCGATCCCGTGCACGGATGTCTCGGGGTCGCTCGGAGATTGAGGTGGGAGATACGCTGCTGCGAGAAGCAGCTC

			GAAGACGCGAATCGTCGCCTGTCTTTGC<br>TTAGGCAGAGAGACTGGGAACTGAAGA<br>GGCTATGA
XP_010048<br>504	Eucgr.C02148.1	*EgLBD10*	ATGGCGTCGTCATCCACGTCCCCGTGCG<br>CCGCCTGCAAGTTCCTCCGCCGCAAGTG<br>CCAGCCCGAGTGCGTGTTTGCGCCCTAC<br>TTCCCACCAGACCAGCCACAGAAGTTT<br>GCCAATGTCCACAAGGTTTTCGGGGCA<br>AGCAACGTGACGAAGCTTCTCAACGAG<br>CTCCACCCCCACCAGCGTGAGGATGCG<br>GTCAATTCACTCGCCTACGAGGCCGACA<br>TGCGCCTGCGGGATCCGGTCTATGGCTG<br>CGTTGGGGTCATCTCCCTCCTCCAGCAC<br>CAGCTCCGCCAGCTCCAGATGGACCTCA<br>CCTGCGCCAAGTCTGAGCTCTCCAAGTA<br>CCAGAGCCTTGGCCTCGCCGCTGGCCA<br>CGGCCTCATCGCCGCTGCCGCGGCCGCG<br>GCCACCACCCACAACCACCCCCAGAAC<br>CTCGGCATCAACCTCCTCGGCCCTGCCA<br>GCTCGCGGGACCACCATCATTACCACCA<br>CCAGTTCTTTCCCCGTGACCACCAGCAC<br>CAGATGATCCGGGGCTTCGACGCGGGCT<br>CCAACTACGACGCAAGCCTTCTCGCCAT<br>GAACGTCTCCGCGAGCATCGGCCAATTG<br>GGCCAGTTCCACCAGCCCAGGGCTGCC<br>GCCGGAGACGATCGCCGGAACATCGAC<br>CCTTCTTAG
XP_010051<br>281	Egrandis_v1_0.<br>048576m.g	*EgLBD11*	ATGCCGACCACCGCCAGCGCCAACAGG<br>CCCGACTCGCGGGCGTGCGCGGCGTGC<br>CGGCACCAGCGCCGGAAGTGCCGTCCC<br>GACTGCCAATGGGCCGAGTTCTTCCCCG<br>CCGCCCTCCCCCAGACCTTCGCCAATGC<br>CCGCTCCCTCTACGGCCTCAGGAACATG<br>AACGAGCTGACCAGGCTCCTCCCGCCC<br>GAGCTCCGCAGGACCGCCATGGAGACC<br>ATCATCTTCGAGTCCAACGTGAGGGCCT<br>TCGACCGCGTCGGCGGGTGCTACCGCAT<br>GATCCGCGATCTGGCTGCCAGGGTCCGG<br>CTGGTGGAGTGCGAGCTCAGGCACACG<br>CATCAGCTGCTCGCCTTGCTTCGCGGCT<br>CGGAGCTTAGTACTGATAACAATGTCGA<br>GGATGACGATCTAGGGTTGGCGCAAAA<br>ATCGAGTGAAGATCAAGGGGTTTTGATG<br>CGTGACTATTTCGTGACGGACGATCTCG

			GAGCGTCGACTTCATCATGTCGAGTCGATGAACCCTCAGGCGCGAATCGTGTTTGCCAAGGCGACCGGCTACTGAATTTTCTTGATGAACAGATAATCAGGTTCGATCCTCAACTTGGCATGCATATATTTCTCTAG
XP_010053892	Egrandis_v1_0.052900m.g	*EgLBD12*	ATGAGCTCAAACCCTAGCTCGAGTGCATCCGCCAGCGGCGAGATCGCCAGCACCGCCGGTGGTGGAAGCGGTGGACCCTGCGGAGCATGCAAGTTCTTGAGGAGGAAGTGCATCACGGGATGTGTTTTTGCGCCCTACTTCGACTCGGAGCAAGGAGCGGCTCATTTCGCGGCAGTCCACAGAGTCTTCGGGGCGAGCAACGCTTCGAAGCTGCTGCTGAACATCCCAGCCCACAGGCGGCAAGACGCTGTCGTGGCGATATGCTACGAGGCGCAAGCTCGCCTTCGAGACCCTGTCTACGGATGCGTCGCCCACATCTTTGCTCTCCAGCAACAGGTGGTAAACCTACAAGCTGAGCTCTCATATCTACAAGCCCATCTTGGACCACTAGAGCTACCGACGCAACCTCTGCCACCCCTTCTTCCAATGCAGTTGCCGGTGGCGCCACATTCTCTCTCTATAGCTGACCTTCCAATTCCGTCGGCCTCGCCATCCATGCCCGCGCAATATGATGTATCGTCACTTTTTGATCCGACGGTGCATCCATCCTCATGGGCTGCTGGCACGCTACTGCGAAGGACAATTGATCCACGTCAATTCAGTGGCGGCTGCTCATTACCAACCACCATGGTCGAAGGAGAGGGTGATCTTCGAGCATTGACAAGTGAATTTCTCAGCACACAATGCCCTCCCCGAATGCCTGATACTTCACCGACGCCGTGCCCCTCTAAAAAAGACAAACCTTGA
XP_010056297	Eucgr.E01414.1	*EgLBD13*	ATGAGTTCTAACCCCAGCTCTAGCGGCGGAGGCGGAGGCGGCGGAGGGGGGAGTGGCAGCGGCGGGGGCAGTGGGAGCGGGAGCGGGAGCGGAGGGCCATGCGGCGCGTGCAAGTTCCTGAGGAGGAAGTGCGTGCCCGGGTGCATATTCGCGCCCTACTTCGACTCGGAGCAAGGGGCGGCGCACTTCGCGGCAGTGCACAAGGTGTTCGGCGCGAGCAACGTGTCGAAGCTCCTCCTCCACATCCCCGTCCACAGGCGGCTCGACGCGGTGGTCACCATATGCTACGAGGCGCAGGCCCGCCTCCGGGACCCCGTCTACGGCTGC

			GTGGCCCACATCTTCGCCCTCCAGCAGCAGGTGGTTAACCTCCAAGCCGAGCTCTCTTACTTACAAGCCCACCTGACGACGTTGGAGCTCCCGGTGCCTTTACCGCCTCCGCCGCCGCCCCCAGCTATGGGGCCGCAGCTATCAATAGCTGATCTCCCATCCGCCTCGTCGATCCCCGCTACTTATGATTTGTCCTCGCTTTTTGATCCAATGGCACAAGCGGCGTGGATGGTGCCCCAACGGCAGATCGATTTCCGCCAATTCGAAGGCAGCTGTGGCGGCGGCGGGTCATCAGCTGCAGGCGGCGGCGATCTTAATGCGCTGGCCCGGGAATTGCTACGGCGGCAAGGATCTCCACCTCCTGGCTGCATAGACGCGATGTCATCGCCATCGTCCCTCTCAAAGTGA
XP_010056298	Eucgr.E01415.1	*EgLBD14*	ATGAGCAATAAAAACGACAATAACACCGCCAGCAACAGCGATCCCAACGGGTCGTCGTCGTCGAGCACCGCAAGCGGCGGCGGTGGCGGCCCGTGCGGCGCGTGCAAGTTCCTGCGGAGGAAGTGCGTGAGGGGCTGCGTTTTCGCGCCCTACTTCGACGCCGACCAGGGCCCCGCCCACTTCGCCGCCGTGCACCGGGTGTTCGGGGCGAGCAACGCCTCCAAGCTCCTCGCCCGGGTCCCCGCCCACCGCCGCCTAGACGCCGTCGTCACCCTCTGCTACGAGGCCCTCGCCCGGGCCCGCGACCCCGTCTACGGCTGCGTCGGCCACATCTTCGCCCTCCAGCAGCAGGTGGTGAATTTGCAAGCCGAGCTAGCCTACGTTCAAGCCCGTCTCGCTTCGCTGCAGCACTTCCCTCTACAGCCACTGCCGCCCCAAAGCCCGCCGCAGTCTGAACTCCAGTCCTCCTCCGACACGGCATTGCTCTCGGAAGTGGTGCCCAACCCGACTCTGCCAAACATGTACTTCAACTCCTATCAGCTTCAAAATGGTGCATTGGCTGGCCTGCCGAACTTTCGCATGAATCTGGCTGAAGAACGGGAGATCGAAGATGCCCAACTGCATTCATTGGCTAGGGTTTTTATCTCTTCGTCCCGACACTTGCCTCCTCCAGGGGTGAGATTCGACGGCTCGAGTCCGGGCAAGAATTTATAG
KCW73537	Eucgr.E02056.1	*EgLBD15*	ATGAAGGAGAGCGGTGGGGGCTGGAAGCAGGGGATGCCGTCGCCGTGCACGTTGTGCAAGCTGCCGCAGAGGAGGTGCGCGC

			AGGACTGCGTGTTCGCCCCGAACTGCCCGGCCGACGAGCCCCACAAGTTCGCCAACGTCCACGAGGTCTTCGGCGCCAGCAATGTCAACAAGATGTTCCAGGTCACGCACCTCATCTCGTCATCCGTATAA
XP_010056712	Eucgr.E02018.1	*EgLBD16*	ATGGAGGAGAGCAGCGAGGGGAGGAACCGGGGGACGCCGTTGGCGTGCGCAGCGTGCAAGCTGCTGCGGAGGAGGTGCATGCAGGACTGCCTGTTTGCCCCGTACTTCCCAGCCAGCGAGCCCCACAAGTTCACCAACGTCCACAAGGTTTTCGGCGCCAGCAATGTCAACAAGATGTTACAGGACCTACCAGAAGACCAGCGAGCAAACACCGTGAGTAGCCTGGTCTACGAAGCCAACGCCCGAGTACGGGACCCGGTCAATGGCTGCGTCGGGGAGATAACGGCTCTCCAGAGCCAGCTCGCCGACAAGATTGCTGAGGTGGAGCGCCTTCAAGTCTTGCTGGAGGCGGAGAAGTCTAATCGCAGCCCCAGCTCGTAA
XP_010056710	Eucgr.E02016.1	*EgLBD17*	ATGAAGGAAAGCGGTGGGAGCCGGAAGCAAGGAACACCATCGCCGTGTGCGGCGTGCAAGCTGCTGCGAAGGAGGTGCGCGCAGGACTGCGTGTTTGCCCCATATTTCCCGGCTGACGAGCCCCACAAGTTCGCCAACGTCCACAAGGTCTTCGGCGCCAGCAATGTCAACAAGATGTTACAGGACCTACCAGTGCACCAGCGTGGGGACGCCGTGAGTAGCATGGTCTATGAAGCCAACGCCCGCGTACGGGACCCGGTCTATGGCTGCGTCGGGGCCATCTCGTCTCTCCAGCAACAAATCGACATGCTTCAGAGCCAGCTCGCCCAGGCCAGGGCTGAGGTGGTGCACCTCCGTGTCTGGCAAAACGCGACCATGTCTAACCATGGCCCGAGCCCAACCAGCCCGAGCAACAGCGGGTCACCGTCATCACGGCTCATGGCCTCCCATGGCAAATCCATTTTTGATATGGACATGATGGTGGACCAACCTAACTTGGGAGACTCAGTCTGGTGA
XP_010056894	Eucgr.E02410.1	*EgLBD18*	ATGGAGTCATCAGATACAGGTGCTTTCGAATCGTCTCCTCCTTCATCTCTAGTTCCTCCGTCTTGGTCGTCTCCGACTCCTGCTCTTCCGCTTCCGGTGGTTGTGGCCAGCCCCTGCGCGGCGTGTAAGACATTAAGGAG

			GAGATGCTCAGAGAGCTGTGTCTTGGCC<br>CCGTACTTCCCTCCCTCAGAGCCCGCCA<br>AGTTTGCCATCGCCCACCGCGTGTTCGG<br>CGCTAGCAACATCGTCAAGTTCCTGCTG<br>ACACTTCCCGAATCGCAAAGAGATGATG<br>CAGTGACCAGCATGATTTACGAGGCCAG<br>TACAAGGATTCGAGACCCTGTCTACGGT<br>TGTGCCGGGGCAATAAGTCAACTCCAAA<br>TGCAAATCAGCCACCTCCACGCACAGCT<br>AGCCAAGTCACAAGCTGAACTCATAAG<br>CATGCAAATCCAACGGGCTAATCTCACG<br>GAACAATTGCAGATGACCAAGTCTCAG<br>GAGTCATTGCCGCAGCTGTCCGTTAAGG<br>GCCTCCTTAATAATCCATACGACTACCAA<br>ATAAACTCATGCTTGGATGATTATAATGG<br>CTTTGGCTCATTTTGGGATCCTCTCTGGA<br>CATGA
XP_010058<br>105	Eucgr.E04355.1	*EgLBD19*	ATGGAGGAGAGCAGCGAGGGGAGGAA<br>CCGGGGGACGCCGTTGGCGTGCGCAGC<br>GTGCAAGCTGCTGCGGAGGAGGTGCAT<br>GCAGGACTGCCTGTTTGCCCCGTACTTC<br>CCAGCCAGCGAGCCCCACAAGTTCACC<br>AACGTCCACAAGGTTTTCGGCGCCAGC<br>AATGTCAACAAGATGTTACAGGACCTAC<br>CAGAAGACCAGCGAGCAAACACCGTGA<br>GTAGCCTGGTCTACGAAGCCAACGCCC<br>GAGTACGGGACCCGGTCAATGGCTGCG<br>TCGGGGAGATAACGGCTCTCCAGAGCC<br>AGCTCGCCGACAAGATTGCTGAGGTGG<br>AGCGCCTTCAAGTCTTGCTGGAGGCGG<br>AGAAGTCTAATCGCAGCCCCAGCTCGTA<br>A
XP_010059<br>846	Eucgr.F00115.1	*EgLBD20*	ATGAGCTGCAACGGCTGCCGGGTCCTTC<br>GAAAGGGTTGCAGCGAGAGCTGCGTGC<br>TGAGGTCGTGCCTGCGGTGGATCCCCTC<br>CCCCGAGGCCCAGGGCAACGCCACCCT<br>CTTCCTCGCCCGCTTCTTCGGCCGCAGC<br>GACCTCCTCTCCCTCGTCGCCGCCGTCC<br>CCGATTCTCAACGCCCCGCTCTGTTTCA<br>AAGCCTGCTGTTCGAGGCGTGCGGGCG<br>CACGGTGAACCCGGTCGGCGGGGCGGT<br>GGGGCTGCTCTCCAGCGGCAACTGGCA<br>CGTGTGCGAGGCGGCGGTCGAGACGGT<br>GCTCGACGGCGGCGTCGTCCAGGCCCT

			CTGCGGCGGCGCCGCCACCCTGGCCTTC GGGCTCGACCAATCCGCCGACTCGCCCT TCTTCCCGCCGAGCACGTGCAGGGGGC AGAGCCTGCAGCAGTCCCTGCAGGTGC CGGCGTCGATCCAGGTGGACGTCAACC TCACCCTCCCCCTCTACGACCCGGACCT CAGCTTCAAGGAGAAGCTGCCCGGGAA GAGACGCGGCAAGAGGAGCCACGTGAC AAATAATTGGCCCACCCGGACGACCAC GACGACCACGGCGGCGGCGACCACCCG CCTGAGCTCCGAGGCGTCGGAGGTGAT CGACCCCCCGAGCGGCTACGATCACGG CGGCGTGGGCCCCGGCGGTCAAGAGAC GAAGCTCCTGAACCTGTTCGTATGA
XP_010061 881	Eucgr.F02492.1	*EgLBD21*	ATGAGGATGAGTTGCAACGGATGCAGA GTGCTGCGGAAGGGCTGCGGCGACGAC TGCAGCATCCGGCCTTGCCTGCAGTGGA TCAAGTCGCCCGACTCCCAAGCCCACG CCACCGTCTTCCTCGCCAAGTTCTACGG CCGCGCCGGCCTCATGAACCTCATCAAC GCCGGCCCCGAACACCTCCGCCCCGCG ATTTTCCGATCCTTGCTATACGAGGCCTG CGGCCGGATCCTGAACCCGATTCACGGG TCGGTCGGGCTGCTCTGGGACGGGAGC TGGCAGCTCTGCCACGCCGCCATGGAA GCCGTCCTCAGCGGCGTGCCGATCACGC CGATCGCCTCGGCCGCCTCCGAGGCCGC CGCGCCGCCCCTGAAGGCCTACGACATC CGGCACATCCCGAGGCAGGAGAACCCC GCCGCCGCCGCGGCGATGATGGCGGCC GCGGGCGAGGCCAACAGGGCGAGGCC GATCCGGTCCGGTCGGTTCAAGCGGCCC GGAGCCCGGACGAAGGCTCAACGGCAC CCGGCGCCGCCGCCCCCGGCGGTCGAG TGGTGTGACGAAGAGTCGAACCGGTCG CCGAGCCACGACTCGTCTTTGAGCCACC AGTCGGTGCTGGGGGCCGCGAACGCGG GGGTCGGGTTCGGCGGCGGCGACAGCA AGGAGACGGAGAGCAACAGCATGGTGT CGGCGGAGACCACGACGACGACAGTGG CCGAGCCCCCTCAGCCGCTCCAGCCCG AGCCGGAGCCGGAGTCGGCGGCTGCGG CGGGGGCGGCCGAACGGAGGACGGA GCATCCGCCGAGCCGGAGAGCGAAGTG

			GGGCTCGAGCTGACGCTAGGGTTCAAG<br>CCGGCGTCACGTGAGGACCACGTGATC<br>CCGGTCAAGAAGAGGAGGTTCGAGCAG<br>AGAGACGAAGCGTGCAGCATGGAGCTG<br>GGGCTTTGA
XP_010064<br>042	Eucgr.F04424.1	*EgLBD22*	ATGAAAGAGAGTGGAAGAAAACAAGG<br>AACGCTGTCGCCATGCGCGGCGTGCAA<br>ACTGCTCAGGAGGAGATGCGCTCGAGA<br>CTGCGTGTTCGCTCCTTACTTCCCAGCT<br>GATCAGCCCCAGAAATTCGCTATCGTTC<br>ACAAGGTCTTCGGTGCCAGCAATGTCA<br>ACAAGATGTTACAGGAATTACTAGAGCA<br>CCAGAGGAGTGATGCGGTGAGCTCCAT<br>GGTGTACGAGGCAAATGCAAGGATGCG<br>AGACCCTGTATATGGCTGCGTTGGGGCC<br>ATATCGTCGCTGCAGCAACAGGTCGACG<br>TGCTTCAGAACCAGCTCGCGCTCGCGC<br>AGGCCGAGGTCGTCCACATGCGCCTGC<br>AAACGCCTCAGTTCCCTTCGACCTCGAG<br>CTCAGCCATCGCGGCCACCAACAACGAT<br>AGCTCGCATTCCAGGCGGTCATATTACC<br>CTTTGAACGGGGTTGAGCAGGCCGATG<br>TCGAGGACTCTTTGTGGTCATGA
XP_010065<br>916	Eucgr.G01261.1	*EgLBD23*	ATGAGCTGCAATGGATGCCGGGTCCTCC<br>GCAAGGGCTGCAGCGACACCTGCATGC<br>TCCGGCAGTGCCTCCAGCTCATCGACTC<br>CCCCCAGGCCCAGGCCCACGCCACCCT<br>CTTCGTCGCCAAGTTCTTCGGCCGCGCC<br>GCCCTCATGTCCTTCGTCTCCGCCGTCC<br>CCGCCTCCCACCGCGCCGCTCTGTTTCA<br>GTCTCTGCTGTTCGAAGCGGTGGGGAG<br>GACGGTGAACCCCGTGGGAGGGGCGGT<br>GGGGCTGATCTGGGCCGGCAACTGGCG<br>GCTCTGCGAGGCGGCGGTCGACACGGT<br>GCTCCGCGGCGGCGCGCCGCACGCGCT<br>CCTCGAGCCCGCGGACGCCGTCCCGTC<br>GTTCGAGCTCGACCGGAGCTCGGTGTC<br>GGAGGCGGACCACTTTAGGCCTCGGAG<br>CCCACCATCGCCGCCGCCACCGCCGCCG<br>CCGCCGCCGCCGCGGCATCCGCAGCCG<br>CCTCGGGACGAGCCCACGAAGGCCGGA<br>GAGGATCGCCTCGACCTGTCCCTGATGT<br>CTCCCGACTTGCGGCGGCGGCGGCGGC<br>GGCGGCCGGCGACCCCATCGGAGGAGG

			GGGAGGAGTCGGAGACGACGACGGCG ACGTGGCGGAGCGGGTCGTCGGAGGGT GGGTGGCCGAGGCAGGACGCGAAGCTC CTGAGACTGTTCGTGTGA
XP_010066 723	Eucgr.G02321.1	*EgLBD24*	ATGAGAATGAGCTGCAACGGCTGCAGA GTCCTCCGCAAGGGCTGCAGCGATGAC TGCGTCATAAGGCCCTGCCTACAATGGA TCAAGTCCGCCGATTCCCAGGCCCACGC CACCGTCTTCCTCGCCAAGTTCTACGGC CGCGCCGGCCTCATCAACCTCATCAATG CCGGCCCTCCCCACCTCCGTCCGGCCAT TTTTAAGTCGCTGCTCTACGAGGCCTGC GGTCGGATCATCAACCCCATATACGGGT CGGTCGGGCTGCTCTGGTCCGGCAACT GGGGGCACTGCCAAGCCGCCGTCGACG CCGTCCTCAGGGGCTCCTCCCACATCAT GCGGCACCCTTCGCTGGATTCAGGTTCG ACCCCGGCCGCGTCTCAGCCCCACCCC GCCCTTAAGGCCTACGACATCCGCCACT TCCCCAAGGCCGGGGGCTCACAAGGCC TCCACAGGGCCCGGTTCAAGCGCCCCA GGCTCGGCGTCGACCTGAAGCCGGACG GCTGGCCTCACCCGGAGGCGAGCTACT CGACATCGCCAGAGGATGACAGCGTGT TCTCCGTGGAGACCGTGGAGGCTGAGC CTGAGGGCCCGTCCCAGCCGAGCCAGA TTTTGGCTTTCGACGGCCAAGGTGGTGA GAGTGACGAGGATCGGGTCGGGTTGGA GCTCACTCTTGGCGTGGCTCAGAGTCAA CAACAGAGCGGCTCATGCAAATCTGAG TCTGATCATGGTCAACACTCGTGA
XP_010068 859		*EgLBD25*	ATGGAGAAGTACGAGGAGTCTCAGAAG CCCCGATCGCTGCCCTCGCCCATCGCCA TCCCTTCCTCTCCTTTCTTCCCCCACTCG CCCACTTACTCCCACTCCCCGCCGTCGC CGTCGCCCCCGCCGCCCCAGCCCCAGC CCCAGTTCTGCCCGGGGACCCCGCCCC ACGACGGCGGCCCCCCGCCGTCCCCGC CCGCGGTGGTCATGAGCCCTTGCGCCGC CTGCAAGATCCTGCGGCGGAGGTGCGC GGACAAGTGCGTCCTGGCCCCCTACTTC CCGCCGACGGAGCCGTACAAGTTCACC ATCGCCCACCGCGTCTTCGGCGCCGCCA ACATCATCAAGTTCTTGCAGGAACTGCC

			GGAGTCCCGGAGGGCCGACGCGGTGAG CAGCATGGTCTACGAGGCGAGCGCGCG GATCCGCGACCCGGTGTACGGCTGCAC GGGCGCCATCTCGCAGCTCCAGAAGCA GGTCGTGGAGCTCCAGGCGCAGCTCGC CAAGGCGCAGGCCGAGGTCGCGAACCT GCAGTGCCAGAACACCAACCTGTTCGC CCTCATCTGCATGGAGATGAGCCAGTCC CAAGACCAGCCCGTCTTCCAGCAGCAG TGCCCCGCTGCGATGGACGCGAGCTGCT TCCCGGACGAGACCAACCTCATCCCTTC TTGGGAGCTTTGGGCGTGA
XP_010067432	Eucgr.G02955.1	*EgLBD26*	ATGACAGGGCTTGGCTCTTCATGTGGTG CATGCAAGTTCCTGAGGAGGAAGTGCA CCAGCGACTGTGTTTTCGCTCCTTATTTC TGCTACGACCAGGCCTCGACCCATTTCG CGGCCGTTCACAAGGTGTTTGGCGCAA GCAATGTGTCCAAGCTGCTGCTGCACCT GCCAGTGCACACCCGGAGTGATGCTGC AATCACCATTTCTTATGAAGCCCTGGCC AGGATGAGAGATCCCATCTATGGTTGCG TGGCTCACATTTTTGCACTCCAGGAGCA GGTGGCTAATCTGCAGGAGGAGATCGA GATTCTAGGGACCCAAATGGCCAACCTT GGAGTTGGGGTTCCCAGTTGGAGAAAT TCACCTGTTCCGGGTAACCCCAGCAATT TTGGTCCACAGTTCTCAGGGAGTGATGC CATGAACGTGCAATTCTATCTGAACCAT CAGCAGGACCCACTTCTTCCTCAGGCA GAAGCTGCTACCACCACCAACCTCCAG GCCTTGGAAAGCTCAATGAACGAAGAG TTCCCTCCTATATATGGTTGGGAGGATCA GAATCCGTTCTGTGACAATTACCCAAAT CTTCTGGAGAGACTCTTTGAAGGTGTGG ACCGTGAGGCCTTCAGCAGTTGTTCATG GCTGAACGGTGGAAATGGTTTAGGAAA GTGA
XP_010067553	Eucgr.G03078.1	*EgLBD27*	ATGAGGGCGATGCGAATGAAGCAAGCA ATGAAGGGTCATGAGCCTCGTTCCAGCT CTTCCTGTGCAGCCTGCAAGCTGTTGAA GAGGAAGTGCACGTCGACGTGCATCTTT GCGCCGTACTTCAGGTCCGACGAGCCG AAGAAGTTCGCCAAGGTGCACAAGGTG TTTGGGGCGAGCAATGTGAGCAAGATC

			CTGGTTGAGGTTCCCGAGGAGCAGCGGGAGGACACGGTGAACTCACTCGCTTACGAGGCCGAGGCAAGGCTGCGGGATCCGGTCTACGGGTGCATCGGGGCGATAGCGCTGCTCCAGAGGAAGATGCTTGAGCTGCAACATGATCTAGCCATTGCTCGGGCCCGTCTCGCTCGATATACCGTGCCTGCAGTGAGCACGAGTTCGCCGAGCGTCTTGATGGATGAGAACGTTGGCATGGTGTCTTTCTATGACATGCCTTGCTGCAGTGGAGTGAATGAGAGTTTCGGCCAGAGTTCATCACAATTGACCCAAGTCCAACCCATGAATGATTTTGGCCAGTTCCCTTACATCTTTTGA
XP_010067560	Eucgr.G03089.1	*EgLBD28*	ATGGGGGGAAACTCGCCTTGTGCATCTTGCAAGTTGCTGAGACGTCGATGCGCTAAAGGCTGCATATTCGCTCCTTATTTTCCTCCAGATGATCCTCACCGTTTCGCCATTGTCCACAAGGTCTTTGGTGCGAGCAACATCAGCAAAATGTTGCAGGAGCTACCAGTGCATCAGAGAGCTGATGCTGTGAGCAGCCTTGTGTACGAAGCCAATGCAAGAGTCCGAGACCCCGTGTACGGATGCGTTGGCGCCATCTCCTACCTCCAAAACCAGGTGGCTCAGCTACAGATGCAGCTCGCTGTGGCTCAGGCCGAGATCCTGTGCATACAAGTCCAGCAAGAGCCCGTGGCCATGCCGTCTCCAGTTGTACCGATGGACAGCTCAGACGACAAGTCGTTCTTCCTCCACAATAACCTCCCTCAGTACTTTAACTTGGCGTCAAACTCCAGCAATGTAACTCATGAGTCTCTCAAGAGAGAGAGCTTCTTTGGACATGACATGGTTTCTTAA
XP_010067591	Eucgr.G03121.1	*EgLBD29*	ATGTCCAGAGAAAGTAGGGAGAGATTTGATGAGATAGGCAAGAAGATCAAAAGAGACGGAGATGCTTCGCATCATCATCTTATGGGAAGGAGACACATGCTGGGTCCTCCAGGAACCCTAAACACCATCACTCCCTGTGCAGCCTGCAAGCTCTTGAGAAGGAGGTGTGCTCAAGAATGTCCTTTCTCTCCTTATTTCTCTCCCCATGAACCCCAGAAATTCGCTTCTGTTCATAAAGTCTTCGGCGCCAGCAATGTCTCCAAGATGCTCATGGAGGTACCTGAGAGCCAAAGAGCTGACGCAGCGAACAGTCTCGTCTACGAAGCGAATGT

			GAGGCTGCGAGACCCAGTTTACGGGTG<br>CATGGGTGCGATTTCGGCTTTACAACAG<br>CAAATTCAGTCCTTGCAAGCTGAGCTAA<br>ACGCGGTGAGGTCCGAAATCCTCAAGT<br>ACAAGTATAGAGAAGCAGGCATCATCAG<br>TCCCACTTCTCATGTGACTTTGCTTTCAC<br>CGGGCACAGTCTCCATTGCCGCGCCGCC<br>TCCCACCCCGCCACCGCAGTCTGCTCCT<br>ACCTCATTGCCGCCTCCTCTCCCGCCTG<br>CCTCCTCGCCTCCTTCCTTGTTTACACA<br>GCCGGGTGGCACAACTGACTACGGCGG<br>CATCTCCAGCGAGAATGTCTCTTATTTTG<br>GTTAA
XP_010070501	Egrandis_v1_0.045772m.g	*EgLBD30*	ATGTCGTCGTCGAACTCTCCTTGCGCCG<br>CGTGCAAGTGCCTCCGCAGGAAGTGCA<br>CCCAGGAGTGCGTCTTCGCCCCCTACTT<br>CCCCGCCGACCAGCCGCAGAAGTTCGC<br>CTACGTACACAAGGTGTTCGGCGCGAG<br>CAACGTGACTAAGCTCCTCAACGAGCT<br>GAACGTCTCGCAGCGCGAGGACGCCGT<br>CAACTCCCTCGCCTATGAGGCTGAGACC<br>CGCCTGCGGGACCCGGTCTACGGCTGC<br>GTCGGCCTCATCTCTATCCTCCAGCACA<br>AGCTCGACCTGCTCCAGAAGGAGCTCG<br>CCTCTGCCAAGAAGGAGCTCGCCACCT<br>ACGTGGGGCCCCACGCGATGATACCCAC<br>CATGATGCAACCCTCTGGAGCTCCGCCG<br>TACATGGGAAATCCTTCGATGTCAGCGG<br>TGGTGCCTTACAACATGATGCCGATGAT<br>GGGAATCCCGACTGCTGCCGCTGCTGGC<br>GGGCCACCGCTAGTCATCAGGGACCAC<br>CAGAGCCAGCAGCAGCAGCAGCAGCAG<br>CAGCAGCAGCAGGCGTTTGATGCGCAG<br>CAACTCGCAGCCGCCGTGGCAGCGAGG<br>GAGCGGCAAGAGATGATTAGGGCTTATG<br>AGCAGCAGCAAATTAGACAGCAGCAAC<br>AGCAACAACTTGAAATCAAGTTCGACG<br>CAAGCGGCTTTGACGGTGTCGGGAACT<br>CGGTGTCTCCGTCCAGGTACAACAACCC<br>CACAAGCAATGTAATCTCTCCACCGCCG<br>GCTTCCCCGTCCCTGGCTTTGGGGAGTT<br>TCGACAATCCGTACCAGATTCAGGCGCA<br>CCAGCTCTCGCTGCAGCCACAACAGGG<br>ACAAGCACAAGATCAGCAAGAGCATGC

			TCAAGAGCAGAGGTCTGGGAGCGAGGAGCGAACCAACAACAACAACGGTGGTCCTTCCTGTTGA
XP_010026421	Egrandis_v1_0.050617m.g	*EgLBD31*	ATGTCGTCGCTGAGCTCCCCGTGTGCTGCGTGCAAGTGCCAACGCCGGAAATGTACCCCTGAGTGCGTGTTCGCCCCCTACTTCCCCGCGGACCAGCCCCAGAAGTTTGCCTATGTCCATGAGGTCTTCGGCGCGAGCAACGTGGCCAAGCTCTTGAACGAGCTCCATGTGTCGCAGCGGGAAGACGCAGCAAACTCCCTCACCTACGAGGCCGAGGTCCGCCTCCGTGATCCCATCTATGGGTGCGTTGGGCTCATCTCCATCCTCCAGCACAAGCTCAACTTGCTGCAGAAGGAGCTCAGCAATGCCAAGAAAGAGCTCGCTGCCTACATGGGACCCCACTCAATGATGCCAGCCACAGTGCAACCTCCTGGGGCGATGTTTCCGGCCGGTAATCCCTTGTCGTCCATGATGGCATTGCACAACACGGTGCCGGTCATCACGACTAGGGGCAAGGGCGACGCACTATGGCATGGTGGAGGGTCGACGTTGGTAGACCAACAACTGTTGCTTGAAGAACATCAACTGGCGGCTATTGCAGCGGCGAGGGAGCAACAAGACATGGGTAGGGTTTGTGAGCAGCAGCAAATTAGGAGGCATCATGATCAACAGCAACTGCAAAGTCAACATGAAACTAAGTATCGCACAAATGGTTCTGACTGTGTTGGGGAATTTGTTTCTGCAATACAGTACCACACCGCAAATAATGCAATTCCTCCAGGCCCGGCTTCGCCATCACTAGCATTGGGTAGTTTCATGAACCCATACCACGAGCAAGCTCATCGTCTCTCGTTGAAGTCGGAACAAGCATGCATCGAAAAACATCAAGAGCAACGATCCGGTGGCATGGAGGGCCAGAATGATGATGTCGGCCTTTCCTGGTGA
XP_010070643	Eucgr.H02293.1	*EgLBD32*	ATGAGCTGCAACGGTTGCAGGGTTCTGAGGAAGGGCTGCAGCGAGTCCTGCATCTTGCGGCCTTGCCTGCAGTGGATCGAGGCCCCCGAGGCCCAGGGCCACGCCACCGTCTTCGTCGCCAAGTTCTTCGGCCGCGCCGGCCTGATGTCCTTCATCTCCGCCGTCCCTGAGACGCAGAGGCCCGCGCTGTTCCAGTCGCTGCTGTTCGAAGCGTGCGGA

			AGGACGGTGAACCCGGTGAACGGGGCG GTGGGACTGCTGTGGACCGGCAACTGG CCCGTCTGCCAGGCTGCGGTGGAGACC GTCCTGCGCGGCGGCTCGCTTCGGGCCA TGCCGGAGCTCCTCGACGGCGGCGGCG GCGGCGGCGGCTCCCTCCCGGCGTCCG ACGAGGCCTCGGAGGCCGGTGACACGT GCGCCGACGCATGGAAGCTCCGCGATC CGAATCCGGGATCCCGATTCTCCACTTC CAGGTCGAGGATCTCCCCGAAGCGCAA GAGGCTCGACGCCGCCGCCGACAACGC TAAGCTCCTACTCCAGCCGATGGATCTC GATCTCCGCCTCACGCCGACGGACTTCC AGGCCAAGACCAAGCCCGCAGTGAAGG CCGAGCACCAGACGCGGCGGCCGGGGA CCCCGTCGATGACTTCGGAGGAGTCCG GCACCACGACGTGCTTCGAGAGCAACA ATAATGTGGGAGAGCCGGAGACGAAGC TCCTCAACTTGTTCGCCTAA
XP_010029135	Eucgr.I01750.1	*EgLBD33*	ATGGCTTCTTCGAGCTATTCCAATCCGCC ATGTGCAGCCTGCAAATTCCTCAGGCGG AAATGCCTGCCTGACTGCGTGTTCGCCC CTTACTTCCCGCCTGAGGAGCCCCAGAA GTTTGCCAACGTCCACAAGATCTTCGGT GCGAGCAATGTGAGCAAGCTCCTCAAC GAAGTCCTCCCCCACCAGAGGGAAGAC GCCGTCAACTCTCTCGCCTATGAGGCCG AGGCCCGCATGAAGGACCCGGTTTACG GCTGCGTGGGGGCGATCTCAGTGCTCCA GCGACAGGTCATCAGGCTACAGAAGGA GCTGGATGCGACGAACGCTGACCTGAT GCGCCACGCGTGCAACGAGATGGCGGG TAACACCTCGCTCCCAGGGCCTAATCCC GGCCACAACTATTCCGGATCACCGAGAG AGTCATCTAGATACAACGAGAACAATCC CGGGTATTATTACCCTCCCAATTATAATC CCTGGTCCGGCGGTCCCAGTGGAGATCC TGATCAGGGAGGAGGAAGTGCTGTCTA A
XP_010030769	Eucgr.I01908.1	*EgLBD34*	ATGACGATCAAAGGAGGGACGAGCCAG GCGTGCGCGGTCTGCAAGTTCCAGCGG CGGAAGTGCCACAAGGAGTGCCCCCTC GCCCCCTACTTCCCGGCCGACCAGCCGA AGATGTTCCAGAACGCGCACCGCCTCTT

			CGGCGTCAGCAACATCACCAAGATCCTC<br>AAGCAGGCCCCGCCGGACCTCAAGCAG<br>GAGGCGATGCAGTCGGTCATCTTCGAGT<br>CCAACATGCGCGCGCAGTACCCCGTCCA<br>CGGCTGCTGCTGGTACATCCGCCGCCTC<br>CTCTGCCAGCTGCACCAGGTGACCGAG<br>GAGCTCCAGCACGTCAACACGCAGATC<br>GCCATGTGCCGGGACCAAGTCTCGGCCT<br>CCGCCTCACCCTCTCACTATCCTCCTCC<br>ACCCGAACTGCAATTGGGGAACGTCCA<br>CGCAGACGCCGTCCCGACCCGACAATC<br>TTTCCCGGGCGTTTTCGTGAACGGGGAA<br>AGTACTAAACAATCTATAATGTCTCAGC<br>AACCATATAATTATGAGCCCGAGAACAC<br>TAGTCACGTCGGGGCGGCTCAGCCGAA<br>CCTGGCTTCATTACCTGAACTTCTAATGA<br>ATTATGGTGCGGACGTGTTCAGCTATGA<br>GGACGTGCCATTTGACACGATCACGATC<br>GATAAGGAGGAGTCGAGCACGGAATCG<br>TCGTTCTCGTTCAAGAACATGACGCATT<br>CCATCGAACACGCCTCGCGCAATGATCT<br>GAAGAATGCAGCCGCAGGATTCAGCCT<br>AAAGAGTGTCAGATAA
KX840005	Egrandis_v1_0.<br>044837m.g	*EgLBD35*	ATGGACGACAATGGAAGTGGAAGCTTA<br>AGCACAACCGCACCATGTGCAGCTTGC<br>AAGCTTTTGGAGAGGAGGTGCGCCCAA<br>AATTGCCCCTTTTCTCTATATTTCTCGCC<br>TCAAGAAACCCACAAGTTCAAATTATTT<br>CATGAAGTTTTTGATGCTAGCAACTTCT<br>TCAAGATCCTCATGATAAACCCTAGCCA<br>AAAAGCTGAAGCTGCAAATAGTCTTGC<br>GTATGAAGCAAGTTTGAGGGACAGAGA<br>TCCTGTTCATGCAAGCATGGGCGTGATC<br>TCTGCCTTGCAAGGCCACATTCAGAGTG<br>CACGGGCCGAGCTCAATGCAGTTAGAG<br>CACAAATGTCAAAGTATAGGGACCAAG<br>CAGGCATTGTCCCTCCTCCCAATGATGG<br>CTTGCTTCTGCCTAACATCGCTGCTTCA<br>ACGGTCGCACACGTCGCCGTCAAAACT<br>GCCTCGTCTCCATCCAAGCTCGCCATCC<br>TCTTCCTCCTCCTCCTCTTCCATGCTCTC<br>ATGGCCAAGCCTTCCGTCCAAAATTAG
XP_010031<br>173	Eucgr.J00175.1	*EgLBD36*	ATGGGCGGGCACTCGCCGTGCGCGTCG<br>TGCAAGTTGCTGAGGCGCCGGTGCGCG

			AAGGACTGCATCTTCGCTCCTTACTTCC<br>CTTCCGACGACCCCCACAAGTTCGCCAT<br>CGTTCACAAGGTCTTTGGTGCTAGCAAC<br>ATTAGCAAGATGTTGCAGGAGCTTCCGG<br>TTTGTCAGAGAGCCGACGCGGTGAGCA<br>GCCTGGTGTATGAAGCGAGCGCGAGGG<br>TCCGGGACCCGGTCTACGGGTGCGTGG<br>GCGCCATCTCCTACTTGCAGAACCAGGT<br>GTCCCAGCTGCAGACGCAGCTCGCGGT<br>GGCCCAAGCGGAGATCATGTGCTTCCAA<br>ATGCAGCAAGACTCGGCGGTGATGCAG<br>CTCCCGCGCATGATGGACGTCCCCGATG<br>ACGACAGGTCGTCGTCGCCGTTCCTCCT<br>ACCGAACAACCTCACCCACTTCCTTAAC<br>TTGTCCCCCGCCTCTTCAGGCAATGTAA<br>CCCACGACCACCCTCTCAAGAGAGAGA<br>GCATTCTCGGGCATGACGTGGTGTCTTA<br>G
XP_010032<br>234	Eucgr.J01123.1	*EgLBD37*	ATGGAAATGGAATACAAGGCGAGAACA<br>ACCACTATACCAGCACCCGTCACAATTC<br>CCACTAAAGTCTTCTTCCCTTACTCAAC<br>ATCATCCTCATCTTCTTCACCGCCACCAC<br>AACCACCATCATCTCAGTCCTCATCTCC<br>AACCACTCCTCCTCCTCATCCTCCCCCT<br>CCTCCTTCTTCCCTTCCCATTACCCCACT<br>GCCGCCGCCGCCGCCGCCGGCAGTCCT<br>CAGCCCGTGTGCCGCCTGCAAGATCCTG<br>CGCCGGCGGTGCGTGGACAAGTGTGTC<br>TTGGCCCCGTACTTCCCGCCCACCGAGC<br>CTTACAAGTTCACCATTGCGCATCGAGT<br>GTTCGGAGCTAGCAACATCATCAAGTTC<br>TTGCAGGAACTTCCAGAACCGCAGAGA<br>GCGGATGCAGTGAGCAGCATGGTGTAC<br>GAGGCCAACGCGAGGATCCGAGACCCG<br>GTCTACGGCTGCGCCGGAGCCATCTGCC<br>ACCTCCAGAAGCAAGTAAGCGATCTCC<br>AAGAGCAGCTCGCCAAGACTCAAGCCG<br>AGCTCGTCAACCTCGAGCTGCAGCGGG<br>TGAACCTCGTCGCGGCATGCATGGAAAT<br>GGCTCAATCCCAAGAGCCGAACATCAAT<br>TTCCAGCAGCAACTCGCCGACTCAACC<br>GGCTTCTTGGAGGATGGCTCGGCTAGCT<br>GGGAATCGATTTACTGGACGTGA
XP_010032	Eucgr.J01501.1	*EgLBD38*	ATGCGGATGAGCTGTAACGGGTGCAGG

636			GTGCTCCGCAAGGGCTGCAGCGAGAGC<br>TGCAGCATCCGGCCCTGCCTCCAGTGGA<br>TCAAGAGCCCCGAGTCCCAGGCCCACG<br>CCACCGTCTTCCTCGCCAAGTTCTACGG<br>CCGCGCCGGCCTCCTCAACCTCCTCAAC<br>GCCGGCCCCGACCACCTCCGCCCTATCT<br>TTAGGTCCTTGCTCTACGAGGCGTGCGG<br>CCGGATCGTGAACCCGATATACGGCTCC<br>GTTGGGCTCCTGTGGTCCGGGAGCTGG<br>CAGCTCTGCCAGGCCGCCGTCGAAGCC<br>GTGCTCAAGGGCGTGCCCATCACCCCGA<br>TCGCCTCCGAGGCGGCGGCCAGCAGTC<br>ACGCCCCGCCCTCCAAGGTCTACGACAT<br>CCGCCACGTCTCCAAGGACGAGACCGC<br>CGCCGCCGTGGCCGATCTCCACCGCGTC<br>AGGGCTCGCTGCCGGTTCAAGCGATCCT<br>TAGCCAAGGCTAGGTCGTCGACGTCGTC<br>CGCGTCGGCGGCGGCGGCGGCGACGGA<br>GTTGGCGCCGGTTCAGGACGAGCCGGC<br>GAAGTCCTCCGACGACGAGCCCAACCG<br>GTCGACGAGCCACGAGTCGTCGCTGAG<br>CCACCAGTCCGAGGTAATCGCCGCGGC<br>GGCGGCGGCCAGCGCGGAAGGCGACA<br>GCAAGGAGACGCTGAGCGCGGTGTCCG<br>AGGAGACTGCCGAGCCGCCGCTACTGT<br>TCCGGGCCGCCGAGCGGGAGCCGGACT<br>TGGCCGATCGCCGGCCGGGCGGGGCTG<br>AGGAGATCGGGCTGGAGCTGACCCTGG<br>GGTTTGAGCCGGTGGCACGTGCCAATC<br>ACGTGGTCCCGGTGAAGAAGAGGAGGT<br>TCGAGGATTGCGGAGCGAGCTCTAAGAT<br>GGAGTTGGGCCTGGACTGCTCGGCCCAT<br>TTAGGCGGAAGCCCAAAATCAATCCTTT<br>GA
XP_010033<br>317	Eucgr.J02237.1	*EgLBD39*	ATGGCTTCATCATCTGGTTACTCCAGCTC<br>TCCGTGTGCTGCTTGCAAGTTCTTGAGG<br>AGAAAGTGCCTGCCTGATTGCATCTTCG<br>CGCCCTACTTCCCTCCTGAGGAGCCCCA<br>GAAGTTCGCCAACGTCCACAAGATATTT<br>GGGGCTAGCAACGTGAGCAAGCTCCTC<br>AATGAGGTCCTTCCCCACCAGAGAGAG<br>GACACCGTCAATTCACTCGCCTACGAGG<br>CCGAGGCAAGGCTCAAAGATCCGGTGT<br>ACGGGTGCGTGGGAGCTATATCAGTGCT

			TCAAAGGCAGGTCATCAGGCTCCAAAA<br>GGAGTTGGACGCGACGAACGCTGATCT<br>CATCCAGTACACCTGTCGCGAGATGCCA<br>AGCACGGTGTCTAGGCCTGTGCATTATC<br>GGAGGAAGAATAATAACAACATGGTTCA<br>AGTTTATGGAGACGGTGGTTTCGATCAG<br>AACTGTGGAGGGTATTATCATCCTAATGC<br>TTGGAGTAATGTCCATGATGGAGATGGC<br>CAAGAGGGAGATGATGGAGGTATGTGA
XP_010033<br>333	Eucgr.J02250.1	*EgLBD40*	ATGGACGGCGGCGGCGTGAGGAGGAGG<br>AGGAGGAGGAGGAGGAGGAGGGCATG<br>TGCTCGCTGCCGGGTAATGAAGAAAAA<br>GTGCTGGAAGGACTGCAGGTGGGCACC<br>GTACTTTCCCGCGGGAGAGCACGAACG<br>CTTCACCATCCTCCACAGGGTCTTTGGC<br>GGTAACATGATAATGGAAATCCTACAGC<br>AAGTACCGAACGACCGGCGAGCAGATG<br>CCATCCATAGCCTCGTCTATGAAGCCAA<br>CGCCCGAGAAAACGACCCAGTCAATGG<br>GCGCCGGGGACAACTCGCCCAGATGAT<br>TGCTGAAGAGGAGCGCCTTCAAGTCCC<br>GCACCAGGCTGATGGATGA
XP_010034<br>810	Egrandis_v1_0.<br>050484m.g	*EgLBD41*	ATGAACTTCTCCTCCCCACGTCCCACCT<br>CAGCATCGACAGCATCCTCCTCTCCTTC<br>CCCCATAACCGCCGCCGCCGCCGCCACC<br>ACCACCTCGACTTCCTCCGCCTCCAAGA<br>GCAGCTCCACCACCCCGGCCTGCGCCG<br>CGTGCAAGTACCAGCGCCGGAAATGCG<br>AGCCCAACTGCCTCCTCGCCCCCTACTT<br>CCCCTATGACCGCCAGCACCAGTTCCTC<br>AACGCCCACAAGCTCTTCGGCGTCAGC<br>AACATCACCAAGGTCATCAAGAACCTC<br>GCCCCGCACGAGAAGGACGAGGCCATG<br>CGCACCATCATCTACCAGTCCGACGCCC<br>GCGCCCACGACCCCGTCCTCGGGTGCTA<br>CCGCATCATCCGCGACCTCCACCGCCAG<br>ATCGAGTTCTGCCAGGCCGAGCTCGAGT<br>TCGTCCTCCGCCAGCTCGAGCTCTGCCG<br>CGCCCAGGCTCAGGCCCAGCAGGAGGC<br>CGCCATGCAGCAGATGATGGATCCCGGG<br>GCGGCGGCGGGGGCTGGGGCGGGGCCG<br>GTCGGCTGCGACATCGTGAGCCGGGGG<br>GATCTGCTGGGGCAGGGGTACGGCTATG<br>TCCAGCCGACCGCCGGCCATCCGGGCC

			AGGAAGTGGAAGGTTATATCAGTAATGG CCAGGACGGGAACGATCAGGCGTCCTT GCAGGATTTCGGTGCATGGGCCATGCAA GTCTCCACGTTCCCGTCGGACTTTCAGA TCAAGCAAGGCTTTGAAGGCGGATGTG ATGATATGAGGCCCATTATGGACATTGCA GACGACAGGAACGATGGCCTCAATTTG GAAGGCGAAATCATCGACGAGAGAAGG TAA
KCW53955	Eucgr.J03160.1	*EgLBD42*	ATGGATCCGACAATATGTGCCGCGTGCA AATATCTGAGAAGAAAATGCCCTCAAGA TTGCATATTGGCTCCTTATTTCCCTTCCT CGAATCCACAAAGATTTGCTTGTGTCCA TAAGATCTTTGGTGCCAGCAACGTCACT AAAATGCTTCAGCAAGTCCCGGGGCATT TACGGGCGGAAGCAGCAGAGTGCATTT CATATGAAGCAACAGCCAGGGTTCAAG ACCCTGTCTATGGATGCGTTGGAATCAT CACTCAACTGCAACAACAAATCACGCA GATTCACAGTGAGATAATGAAGATTAAG GGTGAAATGGTTGCCCATCGATCTGCAC ACCCACAACACCTTCCATCACCACAACC ACAAGGTGAACCTTCCTGGTTCAGTTCC ACTCATCGACAGGAGCAGGACAACTTT CAATCGGACAAGCATCTATCTGGAAACT TCTAA
KX840006	Egrandis_v1_0. 027428m.g	*EgLBD43*	ATGCATAGGCCGATCAACAATGGCATAG GCGGCACGCACGCGGCCTGCGCCTCGT GCAAGCACCAAAGGAAGAAGTGTGACG AAAAGTGCGTGCTGTCTCCTTACTTCCC CGCGGAGAAATCCGTGGAATTCCAAGC CGTGCACGGGGTGTACGGCGTCAGCAA CGTGACCAAGATGGTGCTTGCCGTGCGC GAGCAGGACCGGCGGTGGGTTGCCGAC TCGCTCATCTGGGAGGCCAGGCACCGG CAGCAGGACCCGGTCCACGGCGCGTTC GGGGAGTACCAAAGGATACAAAACGAG CTCCAATACTACAAGAGCTTGCTCCAGA AGCAAGCGATCCAATCGCAACGCTTGC AAGGGGAGATCAGCAAGAGAAGCCACA GCATGGCGCGAAGCTTTGGCAGGTGGA ACAACAAGTGCACGAACGCGATGGCGT TCGACCCTGGGACGAGCATGAATACCGT GGCGAGTTTCGCGGGAGGGATTGCTCAT

			GGCACCGCTATGAATAGTACCTACGCGC<br>GCGACAGCGGCAGCTGCACAATCGATT<br>CGATTCCATATCAGGGGCAGAGTTCAGA<br>AGATTTGAAACAAGAAAGAGAGATTGC<br>TTCTGGCCTCCCACTCCAACAAGAAGTT<br>ATTAATGAGTTTAATCAACACTACTTTCT<br>CTCAGGTAATTTATGCCGTCCGAGTTTTC<br>TATTCAATTCGATTCCCCGATCGCGATAG
KCW47777	Eucgr.K01522.1	*EgLBD44*	ATGGACAGGATGCAAGGTGATCGGCGC<br>AGGGGCCGCAGCACAGCCAGGCGTGCC<br>ATTCCCCCGGGGGAATTGCCGGCTGAGG<br>AGATGACAGCGCCTCCTCCTGGGGCTCC<br>ATGTGGGGCGTGCAAGTTCTTGAGGAG<br>GAAGTGCATCCATGGGTGCATATTCGCG<br>CCCTATTTCGGGTCAGATCAGGGTGCGG<br>CTAAATTCGCCGCCGTGCACATGGTGTT<br>TGGTGCAAGTAATGTGTCCAAGCTGCTC<br>CTTCACATTCCCGTCAACAAGAGGCAG<br>GATGCGGTCGTGACGATCACATATGAAG<br>CCCAGGCAAGGCTGGCAGATCCTGTTTA<br>TGGCTGCGTATCGACCATCCTTGCATTG<br>CAACAACAGGTGGCGACCCTCCAAGCG<br>GAGCTCTCGCTGGTGCAGACGCAGCTG<br>ATGAGCACCCGGTACGCGGTGGCGAGC<br>GCCTTCCAGGCCCCGGAGATGCAGCAG<br>CCCCAGCCGGCGGCGCACCCTTGCCAC<br>CTCCCCATGCTGCAGCCGGAGTACTTCA<br>ACAACTCCTCCGCCACCTCCAACAACA<br>ACTTCATCAACATCAGCAGCTTCGCCGC<br>CGCCGCCGCCGCCGCCGCCGCCACCAA<br>CAACTTCGCCGCCGCCGCCGCCGCGGC<br>GTCCTCTCACACCCTGGAGCCGCTTCCC<br>CAGTTCCCGCACCCGCCCAGGGACGAA<br>GACGAAGGAGAGGACGAAGCGGAGGA<br>GGAGGAGGAGGGGGAAGCCACGTGC<br>CCGCCATTTTCGCGGACGAAATCTTCCG<br>CGGAAGGTGA
XP_010036886	Eucgr.K02222.1	*EgLBD45*	ATGAAGGGTAGCTGCAATGGATGCCGA<br>GTGCTGAGGAAAGCTTGCGGCGACGAC<br>TGCCCCATCCGGCCTTGCCTCCACTGGA<br>TCAAGACCTCCCCCTCCCAATCCAACGC<br>CACCCTCTTCCTCGCCAAGTTCTACGGC<br>CGCGCCGGCCTCCTCAACCTCCTCCTCT<br>CCGGCCCCGCCCACCTCCGCCCTTCGAT

			CTTCAAGTCTCTGCTATACGAAGCGTGC GGGCGGATCGTTAACCCCATCTACGGCT CCGTCGGCTTGCTCTGGTCCGGCAGCTG GCACCTCTGCCAAGCCGCCGTCGACGC CGTCCTCGCCGGCGACCCCATCGCGCGA GTCTCTTCCGAGTCCGCCATCGGCTGCG CCACCCCGCCCCTCGAAGCCGGCGACA TCCGCCACGTGCCCAAGACAAAGCCCT CGGCGGCGCACCCCGATCAGCTCCGCA AGGTCAGGTCCCGCCGCCGCCGGTTCA AGCGCTCGGCCCCGAAGACGAGGCACG AGGAAGCGGAGGAAGCTGGCGGAGAC GGAGCGGATAGCGACGAGCCTGCGAGC CGCGACTCGAGAGGGAGTCGGGGGTCG GCTGCTGACAGAGAGTTGGCGGAGGCG GAAGCCGGGGAGCGAGCCGCCGATGGT GACGACTCCGAGCTGGAGCTGACCTTG AGGTTGGAGCCGAGAAGAGCTAGAAGG GCTTCCGAGGAGCCACAAAGAGATCAG ATCACGGGCGTGTGCGACGGCGACACG CGCGTGCTGAGCTCTGGTCTGGACGTTC GTCGGAGACTCTGA
XP_010037 527	Eucgr.K02809.1	*EgLBD46*	ATGTCTTCATCCAGCTCCTACAACTCCC CCTGTGCTGCCTGCAAATTCCTGAGGAG GAAGTGCATGCCGGGATGCATATTCGCG CCCTACTTCCCCCCAGAGGAGCCTCAGA AGTTCACCAATGTCCACAAGATCTTTGG TGCGAGCAATGTGACCAAGCTGTTAAA CGAGCTCCCCCCACACCAGAGAGAGGA TGCTGTGAACTCGCTAGCTTATGAGGCT GAGGCAAGGGTCCGTGACCCGGTCTAT GGCTGCGTCGGTGCCATCTCCTTCCTTC AGAGGCAGGTCCAAAGGCTCCAGAAGG AGCTTGAAACGGCCAATGCCGATCTCAT CCGATATGCCTGCAACGGGATTCCATCC TCAGCAGCCATTCCCGGGAGCAATAATC CAGTGCAACCTGTTACGCTACGCCAAAG GGCGGCTCCACTTGAGTTTGGTGGTGTT GGGAGGCCAATAGGCAATGAAGGAGGA GGGGCATTTTACCAAGCTCCTCTTCCTC CCTATCCATATCCTTATCCTCCTCTTCCAT GGAATGATAACTTTGGAGGGGACTTCAA TGGAGGAGGAGGAGGAGGCAGCATGTG A

Table S3 The information of *LBD* gene family in *Eucalyptus grandis*

Gene name	Genomic position	ORF (bp)	Protein length (aa)	Mass(Da)	pI	Localization
EgLBD1	Chr01:43293624..43294255	474	157	17508.9	8.75	Nuclear
EgLBD2	Chr02: 3746119..3747107	837	278	24044.3	5.13	Nuclear
EgLBD3	Chr02:23520442..23521264	711	236	25813.7	5.95	Nuclear
EgLBD4	Chr02:44694521..44696196	672	223	24044.3	5.13	Nuclear
EgLBD5	Chr02:50427154..50428075	789	262	28818.6	8.93	Nuclear
EgLBD6	Chr02:59086041..59086876	480	159	17529.9	8.3	Nuclear
EgLBD7	Chr02:59332685..59333586	774	257	27838.5	9.02	Nuclear
EgLBD8	Chr03:14232937..14233876	750	249	28525.7	5.54	Nuclear
EgLBD9	Chr03:14243456..14243806	363	120	13989.1	9.66	Nuclear
EgLBD10	Chr03:39957609..39958253	645	214	23499.4	8.25	Nuclear
EgLBD11	Chr03:61347354..61347959	606	201	22821.8	6.1	Nuclear
EgLBD12	Chr04:38173475..38175872	747	248	26191.9	7.02	Nuclear
EgLBD13	Chr05:14949609..14952874	735	244	24872.2	7.6	Nuclear
EgLBD14	Chr05:14961547..14964939	714	237	25414.6	8.58	Nuclear
EgLBD15	Chr05:27270334..27270612	213	70	7648.8	8.36	Nuclear
EgLBD16	Chr05:27730568..27735405	381	126	13951.8	6.72	Nuclear
EgLBD17	Chr05:27774155..27777868	516	171	18645.1	8.67	Nuclear
EgLBD18	Chr05:34540619..34542263	594	197	21597.5	5.57	Nuclear
EgLBD19	Chr05:75994147..75994410	381	126	13951.8	6.72	Nuclear
EgLBD20	Chr06: 3586954..3587916	708	235	24837.1	7.53	Nuclear
EgLBD21	Chr06:37052494..37053613	936	311	33085.3	6.16	Nuclear
EgLBD22	Chr06:56812127..56814074	489	162	17949.2	8.31	Nuclear
EgLBD23	Chr07:19430222..19431118	672	223	24302.6	8.91	Nuclear
EgLBD24	Chr07:43909845..43910691	738	245	26408.6	6.82	Nuclear
EgLBD25	Chr07:49099412..49101256	678	225	24676.2	6.08	Nuclear
EgLBD26	Chr07:50719546..50720533	669	222	24376.4	5.03	Nuclear
EgLBD27	Chr07:51949711..51950259	549	182	20304.4	8.85	Nuclear

Gene name	Genomic position	ORF (bp)	Protein length (aa)	Mass(Da)	pI	Localization
EgLBD28	Chr07:52024938..52026069	510	169	18685.3	6.99	Nuclear
EgLBD29	Chr07:52311083..52311790	672	223	24165.5	9.01	Nuclear
EgLBD30	Chr08:20732332..20733246	915	304	33347.3	6.31	Nuclear
EgLBD31	Chr08:20738526..20739410	885	294	32355.5	6.38	Nuclear
EgLBD32	Chr08:29295658..29296523	729	242	25868.3	8.08	Nuclear
EgLBD33	Chr09:27445609..27446136	528	175	19096.2	6.41	Nuclear
EgLBD34	Chr09:29046768..29047623	762	253	28280.9	6.79	Nuclear
EgLBD35	Chr10: 1610960..1611943	525	174	18810.6	8.73	Nuclear
EgLBD36	Chr10: 1872293..1873224	525	174	19160.9	6.63	Nuclear
EgLBD37	Chr10:12346751..12348072	687	228	24932.5	5.93	Nuclear
EgLBD38	Chr10:18643984..18645049	936	311	33038.4	7.59	Nuclear
EgLBD39	Chr10:27477243..27477770	528	175	19452.7	6.19	Nuclear
EgLBD40	Chr10:27603439..27603783	345	114	13425.4	10.72	Nuclear
EgLBD41	Chr10:33915425..33916255	831	276	29931.3	5.15	Nuclear
EgLBD42	Chr10:36998560..36999558	450	149	16747.0	8.19	Nuclear
EgLBD43	Chr11:13887164..13887880	717	238	26791.1	9.1	Nuclear
EgLBD44	Chr11:18632942..18633349	693	230	24495.5	5.28	Nuclear
EgLBD45	Chr11:30188061..30189201	780	259	24992.5	9.52	Nuclear
EgLBD46	Chr11:35443749..35444333	585	194	20923.6	8.21	Nuclear

Table S4 Conserved motifs predicted by MEME program in EgLBD proteins.

Motif	Width	Best Possible Match	Valid domain hit for architecture search
1	55	KFANVHKVFGASNVTKMLNEVPEHQREDAVNSLVYEAN ARIRDPVYGCVGHIWYL	DUF260 (domain of unknown function 260)
2	30	TSSPCAACKFLRRKCWKDCIFAPYFPWDEP	DUF260 (domain of unknown function 260)
3	30	QQQVQNLQKELAKAQAELMKYQCQHMNWPM	no hit
4	30	NWHLCQAAVDTVLRGGPITMIAEEADAGGH	no hit
5	30	MSCNGCRVLRKGCSESCILRPCLQWIDSPE	DUF260 (domain of unknown function 260)
6	30	RERFDEIGKKIKREKDATHGDEMGRRHMMG	no hit
7	30	PLKAYDIRHVPKTEGSAALHDIRRKRQRKR	no hit
8	30	KWEIDCCEKQLEDTNRRLSFLRQRDWELKR	no hit
9	40	EIGLELTLGFKPVAREDHVIPVKKRRFEDCDEACKMELGL	no hit
10	30	GIYNNNYDNNINPCLDEYNGFGSFWEPLWT	no hit
11	30	QSQLADKIAEVERLQVLLEAEKSNRSPSSW	no hit
12	30	YDENCPGYYYPYPWNPWNDGFGGDFDDGGG	no hit
13	32	YNNPTNNVIPPGPASPSLALGSFDNPYHEQAH	no hit
14	41	FDDITIDKEECDTESEFSFKDMRHSIEHAGRNDLKNAAACF	no hit
15	31	HQLAAIVAARERQEMIRVCEQQQIRRHHDQQ	no hit
16	36	HCGNGGGVRGFDEQMDVNPKDNVEDALWMNNQGLNY	no hit
17	30	KPPGKRRGKRRRPTTNWMTSEETTTTTAAW	no hit
18	30	PGSSPPSLFTTPGSTWIYGGISYETTSYHY	no hit
19	43	IFSVETVEAEPEGPGKRNRMLKFDWQGEESDEDRVGLELG LGV	no hit
20	33	WAAWMVPRRTIDFRQFEGGCGGGGTMVEGEGDL	no hit

Table S5 Protein-protein interaction prediction for possible functional protein association networks of EgLBD22.

Predicted Functional Partners	*Eucalyptus grandis* Homologous Gene	Partner annotation	score
EXPA14	Eucgr.G02490	expansin A14; Causes loosening and extension of plant cell walls by disrupting non-covalent bonding between cellulose microfibrils and matrix glucans. No enzymatic activity has been found (By similarity)	0.730
LBD13	Eucgr.G03121	LOB domain-containing protein 13	0.733

Table S6 Protein-protein interaction prediction for possible functional protein association networks of EgLBD29.

Predicted Functional Partners	*Eucalyptus grandis* Homologous Gene	Partner annotation	score
F3F9.16	Eucgr.B00034	general regulatory factor 2; Is associated with a DNA binding complex that binds to the G box, a well-characterized cis-acting DNA regulatory element found in plant genes (259 aa)	0.678
AT5G26330	Eucgr.H03540	plastocyanin-like domain-containing protein / putative mavicyanin (187 aa)	0.629
AT1G54790	Eucgr.H04457	GDSL esterase/lipase (408 aa)	0.628
AT1G14240	Eucgr.G02502	GDA1/CD39 nucleoside phosphatase family protein; Catalyzes the hydrolysis of phosphoanhydride bonds of nucleoside tri- and di-phosphates (By similarity) (483 aa)	0.627
AT5G07310	Eucgr.B03559	ethylene-responsive transcription factor ERF115; Probably acts as a transcriptional activator. Binds to the GCC-box pathogenesis-related promoter element. May be involved in the regulation of gene expression by stress factors and by components of stress signal transduction pathways (By similarity) (263 aa)	0.625
AT3G52900	Eucgr.B03885	uncharacterized protein (164 aa)	0.591
AT3G10590	Eucgr.G02591	SANT DNA-binding domain-containing protein (206 aa)	0.588
DSEL	Eucgr.E02710	lipase class 3 family protein; Acylhydrolase that catalyzes the hydrolysis of 1,3-diacylglycerol (1,3-DAG) and 1-monoacylglycerol (1-MAG) at the sn- 1 position. High activity toward 1,3-DAG and 1-MAG, but low activity toward 1,2-diacylglycerol (1,2-DAG) and 1-lysophosphatidylcholine (1-LPC), and no activity toward phosphatidylcholine (PC), monogalactosyldiacylglycerol (MGDG), digalactosyldiacylglycerol (DGDG), triacylglycerol (TAG) and 2-	0.579

		monoacylglycerol (2-MAG). May be involved in the negative regulation of seedling establishment by inhibiting the breakdown, beta-oxidation and mobi [...] (419 aa)	
AT1G70500	Eucgr.H02555	pectin lyase-like protein (468 aa)	0.579
RNS3	Eucgr.G02497	ribonuclease 3; May remobilize phosphate, particularly when cells senesce or when phosphate becomes limiting (222 aa)	0.578

Table S7 Protein-protein interaction prediction for possible functional protein association networks of EgLBD37.

Predicted Functional Partners	*Eucalyptus grandis* Homologous Gene	Partner annotation	score
DFO	Eucgr.B03990	DSB formation (233 aa)	0.598
SEU	Eucgr.F04275	seuss; DNA-binding adapter subunit of the SEU-LUG transcriptional corepressor of the C class floral homeotic gene AGAMOUS during the early stages of floral meristem development. Is part of the A class cadastral complex that define the boundaries between the A and C class homeotic genes expression and function. Interacts together with APETALA2 and LEUNIG to repress AGAMOUS expression (877 aa)	0.544
AT5G61820	Eucgr.K02186	uncharacterized protein (475 aa)	0.436
LUG	Eucgr.C02838	LEUNIG; Transcription repressor subunit of the SEU-LUG transcriptional corepressor of the C class floral homeotic gene AGAMOUS during the early stages of floral meristem development. Is part of the A class cadastral complex that define the boundaries between the A and C class homeotic genes expression and function. Interacts together with APETALA2 and SEUSS to repress AGAMOUS expression. Also plays a role in ovule and pollen development (969 aa)	0.412
